# Structural basis of Mfd-dependent transcription termination

**DOI:** 10.1093/nar/gkaa904

**Published:** 2020-10-17

**Authors:** Jing Shi, Aijia Wen, Minxing Zhao, Sha Jin, Linlin You, Yue Shi, Shuling Dong, Xiaoting Hua, Yu Zhang, Yu Feng

**Affiliations:** Department of Biophysics, Zhejiang University School of Medicine, Hangzhou 310058, China; Department of Pathology, Sir Run Run Shaw Hospital, Zhejiang University School of Medicine, Hangzhou 310058, China; Zhejiang Provincial Key Laboratory of Immunity and Inflammatory diseases, Hangzhou 310058, China; Department of Biophysics, Zhejiang University School of Medicine, Hangzhou 310058, China; Department of Emergency Medicine of the First Affiliated Hospital, Zhejiang University School of Medicine, Hangzhou 310003, China; Department of Biophysics, Zhejiang University School of Medicine, Hangzhou 310058, China; Key Laboratory of Synthetic Biology, CAS Center for Excellence in Molecular Plant Sciences, Shanghai Institute of Plant Physiology and Ecology, Chinese Academy of Sciences, Shanghai 200032, China; University of Chinese Academy of Sciences, Beijing 100049, China; Department of Pathology, Sir Run Run Shaw Hospital, Zhejiang University School of Medicine, Hangzhou 310058, China; School of Chemistry and Life Sciences, Suzhou University of Science and Technology, Suzhou 215009, China; Department of Infectious Disease, Sir Run Run Shaw Hospital, Zhejiang University School of Medicine, Hangzhou 310058, China; Key Laboratory of Microbial Technology and Bioinformatics of Zhejiang Province, Hangzhou 310058, China; Key Laboratory of Synthetic Biology, CAS Center for Excellence in Molecular Plant Sciences, Shanghai Institute of Plant Physiology and Ecology, Chinese Academy of Sciences, Shanghai 200032, China; Department of Biophysics, Zhejiang University School of Medicine, Hangzhou 310058, China; Department of Pathology, Sir Run Run Shaw Hospital, Zhejiang University School of Medicine, Hangzhou 310058, China; Zhejiang Provincial Key Laboratory of Immunity and Inflammatory diseases, Hangzhou 310058, China

## Abstract

Mfd-dependent transcription termination plays an important role in transcription-coupled DNA repair, transcription-replication conflict resolution, and antimicrobial resistance development. Despite extensive studies, the molecular mechanism of Mfd-dependent transcription termination in bacteria remains unclear, with several long-standing puzzles. How Mfd is activated by stalled RNA polymerase (RNAP) and how activated Mfd translocates along the DNA are unknown. Here, we report the single-particle cryo-electron microscopy structures of *T. thermophilus* Mfd-RNAP complex with and without ATPγS. The structures reveal that Mfd undergoes profound conformational changes upon activation, contacts the RNAP β1 domain and its clamp, and pries open the RNAP clamp. These structures provide a foundation for future studies aimed at dissecting the precise mechanism of Mfd-dependent transcription termination and pave the way for rational drug design targeting Mfd for the purpose of tackling the antimicrobial resistance crisis.

## INTRODUCTION

Mfd is a highly conserved ATP-dependent DNA translocase in bacteria. It recognizes stalled RNA polymerase (RNAP) and removes it from DNA, leading to Mfd-dependent transcription termination. Mfd-dependent transcription termination has several physiological roles.

The best-characterized function of Mfd-dependent transcription termination is transcription-coupled DNA repair (TCR), which is a sub-pathway of nucleotide excision repair (NER) (1,2). In TCR, Mfd binds to RNAP stalled at the lesion site, displaces RNAP, and recruits NER machinery to the lesion site (3,4).

Mfd-dependent transcription termination has also been suggested to play important roles other than TCR. For example, replisome and RNA polymerase translocate along the same DNA template, often in opposite directions. These processes routinely interfere with each other and lead to catastrophic effects on genome stability and cell viability (5). Mfd may resolve conflicts between DNA replication and transcription by removing RNAP stalled at the replication fork, facilitating unimpeded replication, and thus reducing possible DNA damage (6,7).

Recent studies have shown that Mfd promotes antibiotic resistance in diverse bacterial species, including *Mycobacterium tuberculosis*, by increasing the mutation rates (8,9). It is proposed that Mfd-dependent transcription termination leads to mutagenic DNA repair through error-prone gap filling. Compared with wild-type strains, Δ*mfd* strains develop antibiotic resistance much slower and to a lower level. Therefore, the combination of Mfd inhibitor and antibiotics may prevent the evolution of antimicrobial resistance.

**Figure 1.**
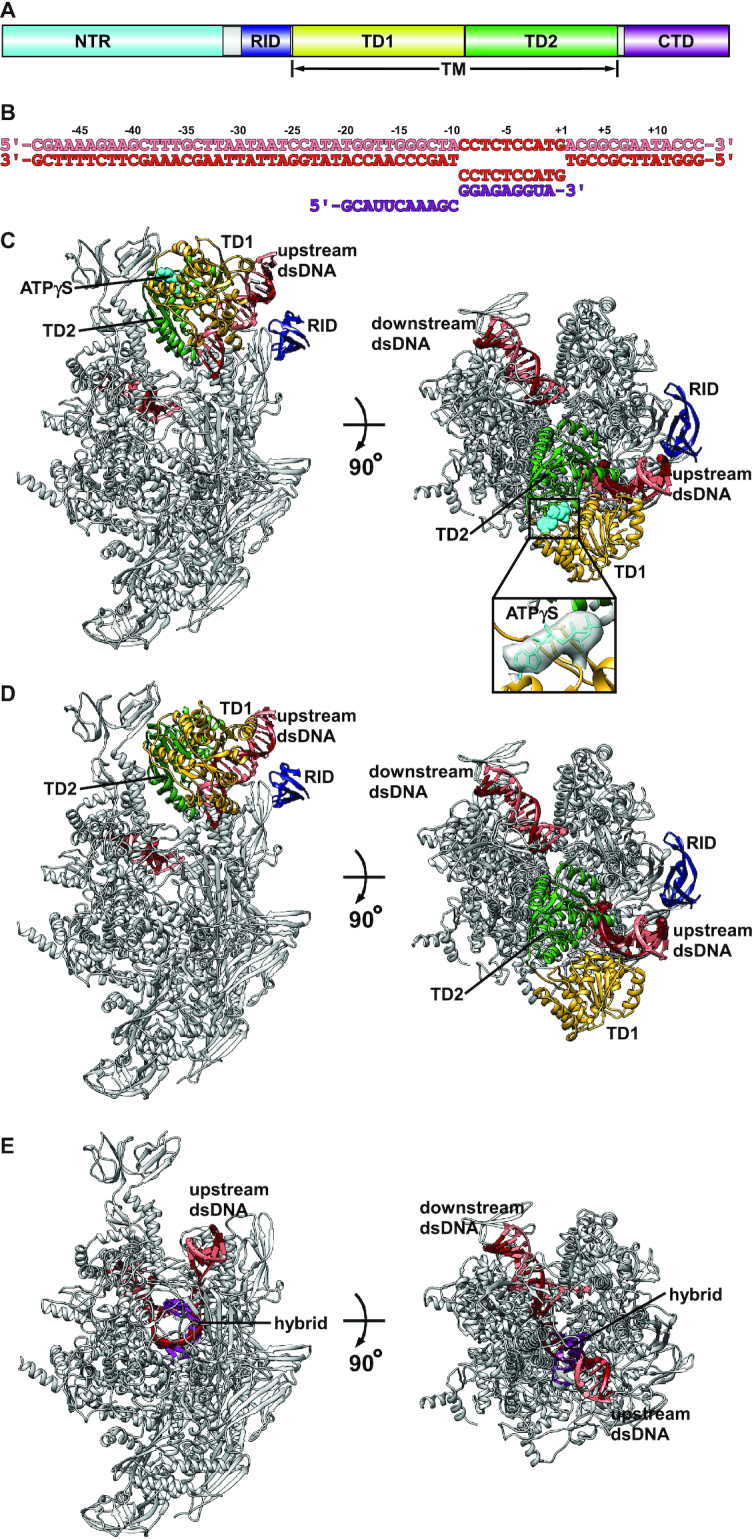
Cryo-EM structures of Mfd-dependent transcription termination complex. (**A**) The schematic representation of Mfd architecture. Cyan, NTR; blue, RID; yellow, TD1; green, TD2; purple, CTD. (**B**) Nucleic-acid scaffold sequence used for cryo-EM. Salmon, nontemplate strand DNA; red, template strand DNA; magenta, RNA. (**C**) The model of MTC^ATPγS^. Protein and nucleic-acid scaffold are shown as ribbon; ATPγS is shown as spheres. Gray, RNAP; red, template strand DNA; salmon, nontemplate strand DNA; blue, RID; yellow, TD1; green, TD2; cyan, ATPγS. The TM binds to the upstream dsDNA and the clamp, while the RID binds to the RNAP β1 domain. The cryo-EM density map and the superimposed model of ATPγS are highlighted in a black box. (**D**) The model of MTC^apo^. View orientations and colors as in (C). The TM binds to the upstream dsDNA and the clamp, while the RID binds to the RNAP β1 domain. (**E**) The model of TEC. View orientations and colors as in (C).

Mfd can be functionally dissected into an N-terminal region (NTR), an RNAP interacting domain (RID), a translocation module (TM), and a C-terminal domain (CTD) (Figure 1A). Biochemical, biophysical and structural analyses have uncovered some aspects of the mechanism of Mfd-dependent transcription termination. First, the interaction between the RID and RNAP β1 domain has been identified to be essential for the function of Mfd (10–13). Second, Mfd can rescue backtracked RNAP by promoting forward translocation via ATP hydrolysis (10). Third, Mfd simultaneously interacts with RNAP via the RID and with DNA via the TM, allowing its translocase activity to generate positive torque on the DNA, thereby overwinding the transcription bubble and disrupting the transcription elongation complex (TEC) (12,14–21).

In the crystal structures of Mfd (11), the conformation of the TM is incompatible with DNA binding and ATP hydrolysis, and the determinants of NTR, which are responsible for recruiting NER machinery, are masked by the CTD. Genetic and biochemical studies suggested that large conformational changes are expected upon Mfd activation (17–19,22,23). To determine the exact conformational changes upon Mfd activation, we solved the single-particle cryo-electron microscopy (cryo-EM) structures of Mfd-RNAP complexes with and without ATPγS. The structures trap the active conformation of Mfd and define the protein-protein and protein-DNA interactions that mediate Mfd-dependent transcription termination.

## MATERIALS AND METHODS

### Protein expression and purification

*Escherichia coli* RNAP-σ^70^ holoenzyme was purified and assembled as previously described (24). *T. thermophilus* RNAP core enzyme and *T. thermophilus* RNAP-σ^A^ holoenzyme were purified and assembled as reported (25). NusG was purified as reported (26).

*Escherichia coli* strain BL21(DE3) (Invitrogen, Inc.) was transformed with plasmid pET28a-NH-EcoMfd encoding N-hexahistidine-tagged *E. coli* Mfd under the control of T7 promoter. Single colonies of the resulting transformants were used to inoculate 1 l LB broth containing 50 μg/ml kanamycin, cultures were incubated at 37°C with shaking until OD_600_ = 0.6. Protein expression was induced by addition of IPTG to 1 mM, and cultures were incubated 4 h at 30°C. Then cells were harvested by centrifugation (5000 rpm; 10 min at 4°C), resuspended in 20 ml buffer A (50 mM Tris–HCl, pH 8.0, 0.1 M NaCl, 5% glycerol) and lysed using a JN-02C cell disrupter (JNBIO, Inc.). The lysate was centrifuged (12 000 rpm; 45 min at 4°C), and the supernatant was loaded onto a 2 ml column of Ni-NTA agarose (Qiagen, Inc.) equilibrated with buffer A. The column was washed with 10 ml buffer A containing 0.04 M imidazole and eluted with 10 ml buffer A containing 0.5 M imidazole. The sample was further purified by anion-exchange chromatography on a Mono Q 10/100 GL column (GE Healthcare, Inc.; 160 ml linear gradient of 0.1–1 M NaCl in buffer A). Fractions containing *E. coli* Mfd were pooled and stored at –80°C. *E. coli* Mfd derivatives were expressed and purified in the same way as wild type protein. Yields were ∼10 mg/l, and purities were >95%.

*Escherichia coli* strain BL21(DE3) (Invitrogen, Inc.) was transformed with plasmid pET28a-NH-TthMfd encoding N-hexahistidine-tagged *T. thermophilus* Mfd under the control of T7 promoter. Single colonies of the resulting transformants were used to inoculate 1 l LB broth containing 50 μg/ml kanamycin, cultures were incubated at 37°C with shaking until OD_600_ = 0.6. Protein expression was induced by addition of IPTG to 1 mM, and cultures were incubated 4 h at 30°C. Then cells were harvested by centrifugation (5000 rpm; 10 min at 4°C), resuspended in 20 ml buffer B (50 mM Tris–HCl, pH 8.0, 0.2 M NaCl, 5% glycerol) and lysed using a JN-02C cell disrupter (JNBIO, Inc.). The lysate was centrifuged (12 000 rpm; 45 min at 4°C), and the supernatant was loaded onto a 2 ml column of Ni-NTA agarose (Qiagen, Inc.) equilibrated with buffer B. The column was washed with 10 ml buffer B containing 0.1 M imidazole and eluted with 10 ml buffer B containing 0.5 M imidazole. The eluate was loaded onto a 5 ml column of HiTrap Heparin HP (GE Healthcare, Inc.) equilibrated in buffer B and eluted with a 100 ml linear gradient of 0.2–1 M NaCl in buffer B. Fractions containing *T. thermophilus* Mfd were pooled and stored at –80°C. *T. thermophilus* Mfd derivatives were expressed and purified in the same way as wild type protein. Yields were ∼2 mg/l, and purities were >95%.

### ATPase activity assay

ATPase activity assays were performed in a 96-well microplate format using a commercial kit (MAK113, Sigma-Aldrich, Inc.). Reaction mixtures contained (40 μl): 5 μM *T. thermophilus* Mfd, 0.4–2 mM ATP, 50 mM Tris–HCl (pH 7.9), 0.1 M KCl, 10 mM MgCl_2_, 1 mM DTT and 5% glycerol. Reaction mixtures were incubated 60 min at 37°C. 200 μl reagent (MAK113A, Sigma-Aldrich, Inc.) was added to terminate the enzyme reaction and generate the colorimetric product. The absorbance at 620 nm was measured using a SpectraMax M5 microplate reader (Molecular Devices, Inc.). Phosphate standard solution was used to calculate the extinction coefficient of the colorimetric product. Less than 2% ATP was consumed to make sure initial velocity was measured.

### RNAP displacement assays

Template strand DNA oligonucleotide and nontemplate strand DNA oligonucleotide (Sangon Biotech, Inc.) were annealed at a 1:1 ratio in 10 mM Tris–HCl, pH 7.9, 0.2 M NaCl and stored at −80°C.

*T. thermophilus* RNAP displacement assay was performed in reaction mixtures containing (20 μl): 2 μM RNAP holo enzyme, 0.1 μM DNA scaffold, 2 mM ATP, 0.2 mM UTP, 0.2 mM GTP, 50 mM Tris–HCl (pH 7.9), 0.1 M KCl, 10 mM MgCl_2_, 1 mM DTT and 5% glycerol. Reaction mixtures were incubated 10 min at 65°C, supplemented with 0.1 mg/ml heparin and 4 μM Mfd or Mfd derivative, incubated 10 min at 65°C.

*E. coli* RNAP displacement assay was performed in reaction mixtures containing (20 μl): 0.1 μM RNAP holo enzyme, 0.13 μM DNA scaffold, 2 mM ATP, 0.2 mM UTP, 0.2 mM GTP, 50 mM Tris–HCl (pH 7.9), 0.1 M KCl, 10 mM MgCl_2_, 1 mM DTT and 5% glycerol. Reaction mixtures were incubated 10 min at 37°C, supplemented with 0.1 mg/ml heparin and 1 μM Mfd or Mfd derivative, incubated 10 min at 37°C.

The reaction mixtures were applied to 5% polyacrylamide slab gels (29:1 acrylamide/bisacrylamide), electrophoresed in 90 mM Tris-borate, pH 8.0 and 0.2 mM EDTA, stained with 4S Red Plus Nucleic Acid Stain (Sangon Biotech, Inc.) according to the procedure of the manufacturer.

### Assembly of Mfd-dependent transcription termination complex

DNA oligonucleotides and RNA oligonucleotide (sequences in Figure 1B) (Sangon Biotech, Inc.) were dissolved in nuclease-free water to ∼1 mM and stored at -80°C. Template strand DNA, nontemplate strand DNA, and RNA were annealed at a 1:1:1 ratio in 10 mM Tris–HCl, pH 7.9, 0.2 M NaCl and stored at −80°C. MTC^ATPγS^ was prepared in reaction mixtures containing (31 μl): 14 μM *T. thermophilus* RNAP core enzyme, 16 μM nucleic acid scaffold, 17 μM *T. thermophilus* Mfd, 5 mM MgCl_2_ and 2 mM ATPγS. MTC^apo^ was prepared in reaction mixtures containing (31 μl): 18 μM *T. thermophilus* RNAP core enzyme, 19 μM nucleic acid scaffold, 21 μM *T. thermophilus* Mfd, 5 mM MgCl_2_. *T. thermophilus* RNAP core enzyme was incubated with nucleic acid scaffold for 10 min at 4°C, and incubated with *T. thermophilus* Mfd for 10 min at 4°C.

### Cryo-EM grid preparation

Immediately before freezing, 8 mM CHAPSO was added to the sample. C-flat grids (CF-1.2/1.3-4C; Protochips, Inc.) were glow-discharged for 60 s at 15 mA prior to the application of 3 μl of the complex, then plunge-frozen in liquid ethane using a Vitrobot (FEI, Inc.) with 95% chamber humidity at 10°C.

### Cryo-EM data acquisition and processing

In the preliminary experiment, some data of *E. coli* Mfd complex with ATPγS were collected, but there is no density for Mfd in the final map. Then we turned to determine the structure of *T. thermophilus* Mfd complex. The grids were imaged using a 300 kV Titan Krios (FEI, Inc.) equipped with a K2 Summit direct electron detector (Gatan, Inc.). Images were recorded with Serial EM (27) in counting mode with a physical pixel size of 1.307 Å and a defocus range of 1.5–2.5 μm. 4266 images and 4035 images were recorded for MTC^ATPγS^ and MTC^apo^, respectively. Data were collected with a dose of 10 e/pixel/s. Images were recorded with a 10 s exposure and 0.25 s subframes to give a total dose of 59 e/Å^2^. Subframes were aligned and summed using MotionCor2 (28). The contrast transfer function was estimated for each summed image using CTFFIND4 (29). From the summed images, 661,783 particles (MTC^ATPγS^) and 1 149 168 particles (MTC^apo^) were auto-picked, extracted with a box size of 200 pixels, and subjected to 2D classification in RELION (30). Poorly populated classes were removed. These particles were 3D classified in RELION using a map of *E. coli* TEC (EMD-8585) (31) low-pass filtered to 40 Å resolution as a reference. The best-resolved classes were 3D auto-refined and post-processed in RELION. The final numbers of particles are 60 650 (MTC^ATPγS^), 24 037 (MTC^apo^) and 558 003 (TEC).

### Cryo-EM model building and refinement

The homology model of *T. thermophilus* Mfd was generated on Phyre2 server (32). The model of RNAP core enzyme from the structure of *T. thermophilus* RPo (PDB 4G7H) (33) and the homology model of *T. thermophilus* Mfd were fitted into the cryo-EM density map using Chimera (34). The model of nucleic acids was built manually in Coot (35). The coordinates were real-space refined with secondary structure restraints in Phenix (36).

### Fluorescence polarization assays of Mfd-DNA interaction

5′ 6-FAM labeled DNA oligonucleotide (5′-AGCAAAGCTTCTTT-3′, Sangon Biotech, Inc.) and unmodified DNA oligonucleotide (5′-AAAGAAGCTTTGCT-3′, Sangon Biotech, Inc.) were annealed at a 1:1 ratio in 10 mM HEPES, pH 7.5, 50 mM KCl. Equilibrium fluorescence polarization assays were performed in a 96-well microplate format. Reaction mixtures contained (100 μl): 0–16 μM Mfd or Mfd derivative, 0.1 μM 6-FAM-labelled DNA scaffold, 10 mM HEPES, pH 7.5, 50 mM KCl, 5 mM MgCl_2_ and 2 mM ATPγS. Following incubation mixtures for 10 min at 25°C, fluorescence emission intensities were measured using a SpectraMax M5 microplate reader (Molecular Devices, Inc.; excitation wavelength = 494 nm; emission wavelength = 518 nm). Fluorescence polarization was calculated using:

(1) $$\begin{equation*}{{P}} = ({I_{{\rm VV}}} - {I_{{\rm VH}}})/({I_{{\rm VV}}} + {I_{VH}})\end{equation*}$$

where *I*_VV_ and *I*_VH_ are fluorescence intensities with the excitation polarizer at the vertical position and the emission polarizer at, respectively, the vertical position and the horizontal position.

Equilibrium dissociation constant, *K*_D_, were extracted by non-linear regression using the equation:

(2) $$\begin{equation*}{{P}} = {{{P}}_{\rm{f}}} + \left\{ {({{{P}}_{\rm{b}}} - {{{P}}_{\rm{f}}}){\times}[{{M}}]/({K_{\rm D}} + [{{M}}])} \right\}\end{equation*}$$

where *P* is the fluorescence polarization at a given concentration of Mfd, *P*_f_ is the fluorescence polarization for free 6-FAM-labeled DNA scaffold, *P*_b_ is the fluorescence polarization for bound 6-FAM-labeled DNA scaffold, and [M] is the concentration of Mfd or Mfd derivative.

## RESULTS

### Overall structures of Mfd-dependent transcription termination complex

Because the interaction between *E. coli* RNAP and Mfd is transient, the initial attempt to determine the structure of *E. coli* Mfd-dependent transcription termination complex (MTC) failed. In the preliminary experiment, some data of *E. coli* MTC with ATPγS were collected, but there is no density for Mfd in the final map. Then we turned to determine the structure of *T. thermophilus* MTC. The RID and the TM are highly conserved between *E. coli* Mfd and *T. thermophilus* Mfd (Supplementary Figure S1). ATPase activity assay verified that *T. thermophilus* Mfd hydrolyzed ATP, while substitution of a conserved Walker B residue, E572, affected ATP hydrolysis, but not ATP binding (Supplementary Figure S2A, B) (17). Electrophoretic mobility shift assay (EMSA) confirmed that *T. thermophilus* Mfd displaced RNAP stalled by NTP starvation, while substitution of residue E572, which is deficient in ATP hydrolysis, failed to displace stalled RNAP (Supplementary Figure S2C, D).

To obtain the structure of *T. thermophilus* MTC, we modified the scaffold, which has been used for structure determination of a TEC (31), by extending the upstream dsDNA from 6 bp to 40 bp (Figure 1B), which is necessary and sufficient for Mfd to function (10,17). The cryo-EM structures of MTC with and without ATPγS (MTC^ATPγS^ and MTC^apo^) were determined at 4.1 and 5.0 Å, respectively (Figure 1C and D, Supplementary Figures S3–S9, Supplementary Table S1). As expected, a clear density feature for ATPγS is observed in MTC^ATPγS^ (Figure 1C). The conformations of RNAP in both structures are similar with a root-mean-square deviation (RMSD) of 0.85 Å (2954 Cαs aligned). Although full-length Mfd was used, cryo-EM densities for only the RID and the TM were observed (Supplementary Figure S7). The RID binds to the RNAP β1 domain, while the TM binds to the upstream dsDNA and the clamp. Another class from the dataset without ATPγS was determined at 3.1 Å, lacked the density for Mfd, and turned out to be a regular TEC (Figure 1E, Supplementary Figures S5, S7 and S8, Supplementary Table S1).

### Mfd undergoes profound conformational changes upon activation

The TM, composed of translocation domain 1 (TD1) and translocation domain 2 (TD2), contains the characteristic motifs that identify Mfd as a RecG-like SF2 helicase (Supplementary Figure S1). The TRG (*translocation in RecG*) motif from TD2 is highly conserved among RecG-like SF2 helicases. Its antiparallel helical hairpin conformation is critical for coupling nucleotide hydrolysis to duplex translocation (16,37).

**Figure 2.**
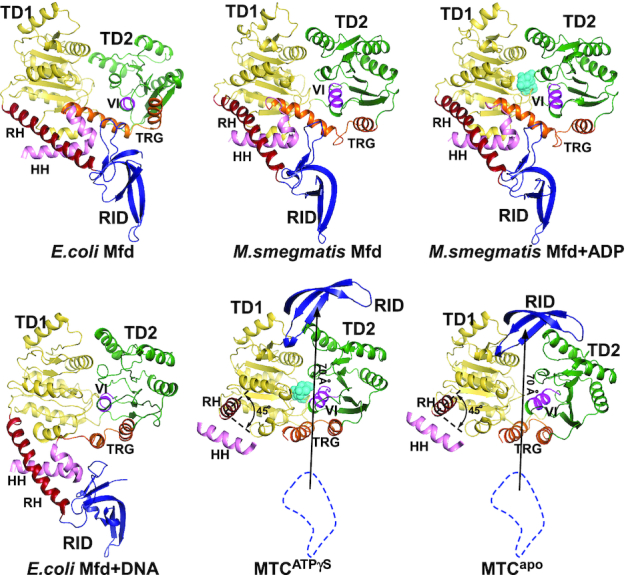
Mfd undergoes large conformational changes upon activation. Top left, the crystal structure of *E. coli* Mfd (PDB: 2EYQ); top middle, the crystal structure of *M. smegmatis* Mfd (PDB: 6AC8); top right, the crystal structure of *M. smegmatis* Mfd in complex with ADP (PDB: 6ACX); bottom left, the cryo-EM structure of *E. coli* Mfd in complex with DNA (PDB: 6XEO); bottom middle, the cryo-EM structure of MTC^ATPγS^; bottom right, the cryo-EM structure of MTC^apo^. The views are aligned so that TD1 of each molecule is in the identical orientation. Yellow, TD1; green, TD2; blue, RID; cyan, ADP and ATPγS; red, RH; pink, HH; orange, TRG motif; magenta, motif VI. The two helices of the TRG motif are almost perpendicular to each other in the absence of DNA, while they are almost antiparallel in the presence of DNA. In MTC^ATPγS^ and MTC^apo^, the RID translates ∼70 Å relative to its position in the absence of RNAP.

The structures of Mfd from different species (*E. coli*, *M. smegmatis*, *M. tuberculosis* and *T. thermophilus*) have been solved in complex with different partners (nucleotide, DNA, and RNAP) using different methods (crystallography and cryo-EM) (Figure 2) (11,13,17,19,38,39). The most striking differences among these structures are the conformational change of the TRG motif and the repositioning of the RID. The two helices of the TRG motif are almost perpendicular to each other in all structures without DNA, while they are antiparallel in all structures with DNA, hinting that DNA is required for the active conformation of the TRG motif. The RID is connected to the TM through a long helix, the relay helix (RH). The RH is in a similar orientation and the RID is in a similar position in all structures without RNAP. Compared with the cryo-EM structure of Mfd in the absence of RNAP, the RH rotates ∼45° and the RID translates ∼70 Å in MTC^ATPγS^ and MTC^apo^, indicating that RNAP induces domain repositioning.

### The upstream dsDNA binds to a positively charged groove of TM

**Figure 3.**
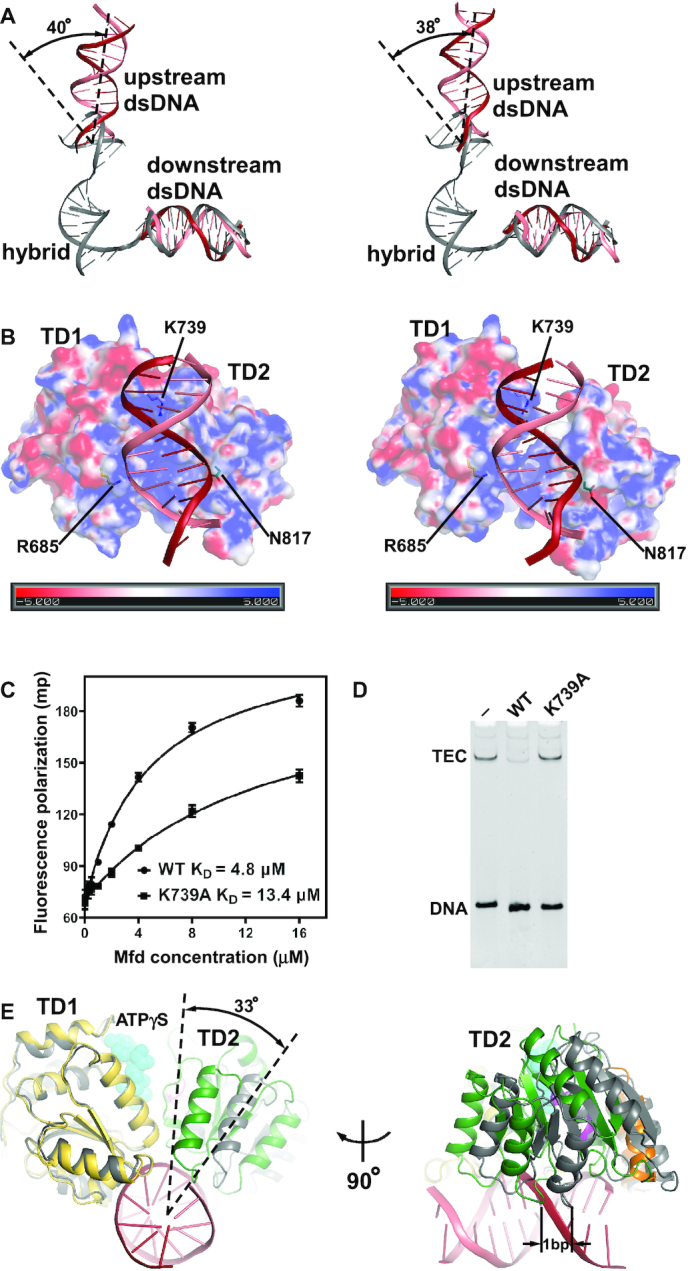
The upstream dsDNA binds to a positively charged groove of TM. (**A**) The upstream dsDNA (salmon, nontemplate strand DNA; red, template strand DNA) bends ∼40° relative to its direction in TEC (gray). Left, cryo-EM structure of MTC^ATPγS^; right, cryo-EM structure of MTC^apo^. (**B**) The upstream dsDNA binds to a positively charged groove between TD1 and TD2. Left, cryo-EM structure of MTC^ATPγS^; right, cryo-EM structure of MTC^apo^. Protein is shown as surface colored according to the electrostatic surface potential (red, –5 kT; blue, +5 kT); nucleic-acid scaffold is shown as ribbon (salmon, nontemplate strand DNA; red, template strand DNA). Mfd residues are numbered as in *E. coli* Mfd. (**C**) Effect on DNA binding affinity of substituting *E. coli* Mfd residue K739. Fluorescence polarization assay in the presence of 2 mM ATPγS. Error bars represent mean ± SD out of *n* = 3 experiments. (**D**) Effect on RNAP displacement of alanine substitution of *E. coli* Mfd residue K739. (**E**) TD2 undergoes a rotation relative to TD1 upon ATPγS binding. The structures of MTC with and without ATPγS are superimposed on TD1. Protein and nucleic-acid scaffold are shown as ribbon; ATPγS is shown as spheres. The structure of MTC^ATPγS^ is colored as follows: salmon, nontemplate strand DNA; red, template strand DNA; yellow, TD1; green, TD2; cyan, ATPγS; orange, TRG motif; magenta, motif VI. The structure of MTC^apo^ is colored gray.

The upstream dsDNA bends ∼40° relative to its orientation in TEC and binds to a positively charged groove between TD1 and TD2 (Figure 3A, B). There is a kink of 29° in MTC^ATPγS^ and 36° in MTC^apo^ at position –16 (Supplementary Figure S10), which is consistent with the single molecule observation (15). Alanine substitution of a conserved basic residue (K739 in *E. coli* Mfd), which is positioned near the DNA backbone, shows defects in DNA binding assay and RNAP displacement assay (Figure 3C, D), verifying that the cryo-EM structures are biologically relevant. Furthermore, alanine substitutions of R685 and N817 in *E. coli* Mfd, which are positioned near the DNA backbones as well, showed severely impaired binding affinity to DNA in a previous report (40).

### TD2 undergoes a rotation relative to TD1 upon ATPγS binding

Compared to MTC^apo^, TD2 rotates by ∼33° toward TD1 in MTC^ATPγS^ (Figure 3E and Supplementary Figure S9B). Structural comparison of MTC with and without ATPγS suggests a model for how ATP binding and hydrolysis result in Mfd translocation (Supplementary Movie S1). The TM first binds DNA in an open conformation. ATP binding then leads to the closure of TD2. Finally, ATP hydrolysis and ADP dissociation reset Mfd in an open conformation at the new DNA register. Superposition of TD1 with and without ATPγS results in different registers of TD2, which are offset along the DNA by ∼1 bp in the direction of translocation (Figure 3E), suggesting that the step size of Mfd is 1 bp.

### Mfd contacts the RNAP β1 domain and the clamp

The interactions between Mfd and RNAP are essentially the same in MTC with and without ATPγS. The interactions in MTC^ATPγS^ will be discussed in the following sections due to its superior resolution.

**Figure 4.**
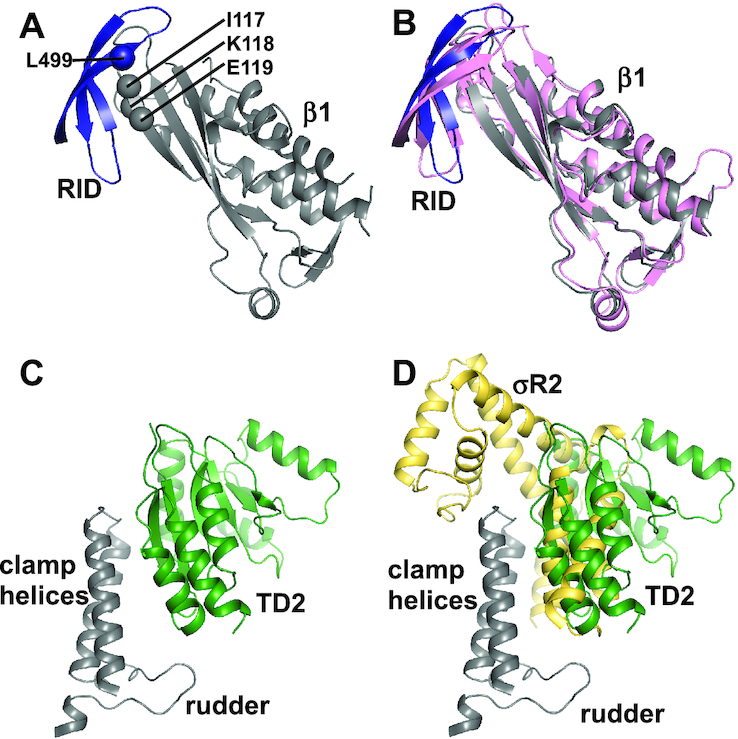
Mfd contacts the RNAP β1 domain and the clamp. (**A**) The RID binds to the RNAP β1 domain. Gray, the RNAP β1 domain; blue, RID. The α-carbon atoms of residues, which have been confirmed to impair Mfd's function, are shown as spheres. Residues are numbered as in *E. coli*. (**B**) Superimposition of MTC^ATPγS^ (colored as in (A)) on the crystal structure of RID-β1 (PDB 3MLQ, pink). (**C**) TD2 binds to the RNAP clamp. Gray, the clamp helices and the rudder; green, TD2. (**D**) σ binds to the clamp helices and excludes the accessibility of the clamp helices. The structure of RNAP holoenzyme (PDB 4G7H) and the structure of MTC^ATPγS^ are superimposed on the clamp. Gray, the clamp helices and the rudder; green, TD2; yellow, σ conserved region σR2.

The RID binds to the RNAP β1 domain with a buried surface area of ∼579 Å^2^ (Figure 4A and Supplementary Figure S9C). The interface has been genetically, biochemically, and structurally characterized (11–13). The structure of the RID and the RNAP β1 domain in MTC^ATPγS^ is superimposable on the crystal structure of RID complexed with the RNAP β1 domain (Figure 4B) (13), indicating that the RID makes a similar set of interactions in both structures. Consistently, substitutions of interface residues disrupt the Mfd-RNAP interaction and cause defects in the RNAP release activity of Mfd (11,12).

TD2 binds to the clamp with a buried surface area of ∼580 Å^2^ (Figure 4C and Supplementary Figure S9D). Specifically, it interacts with the evolutionarily conserved clamp helices and rudder. Transcription initiation factor σ binds to the clamp helices with high affinity (Figure 4D) (33), which would exclude the accessibility of the clamp helices. Therefore, transcript release by Mfd is inhibited by σ (10). NusG binds to the clamp helices, as well (41). Due to its lower affinity, NusG does not interfere with Mfd in RNAP displacement assay (Supplementary Figure S11), which is expected considering the clamp helices of most elongating RNAP are pre-occupied by NusG *in vivo* (42).

### The clamp is open in Mfd-dependent transcription termination complex

**Figure 5.**
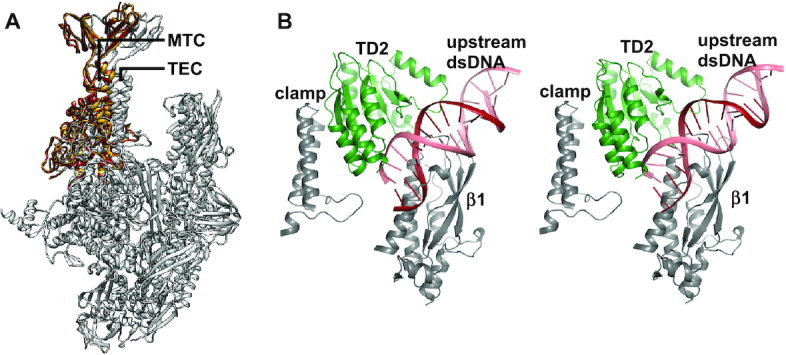
The clamp is open in Mfd-dependent transcription termination complex. (**A**) RNAP clamp conformational change for the three cryo-EM structures determined in this work. TEC structure is used as a reference to superimpose MTC structures via α-carbon atoms of the RNAP core module, revealing a common RNAP core module but with a rotation of the clamp. Gray, the RNAP of TEC; yellow, the open clamp of MTC^ATPγS^; red, the open clamp of MTC^apo^. (**B**) Mfd cannot bind to the clamp and the upstream dsDNA in the same way as in MTC if the clamp is closed. TEC structure is used as a reference to superimpose MTC structures (left, MTC^ATPγS^; right, MTC^apo^) via α-carbon atoms of the clamp, revealing severe clashes between the upstream dsDNA and the RNAP β1 domain. Gray, the clamp and the RNAP β1 domain of TEC; green, TD2 of MTC; red, template strand DNA of MTC; salmon, nontemplate strand DNA of MTC.

The RNAP is like a crab claw with two pincers (43). The clamp, a mobile structural module that makes up much of one pincer, undergoes swing motions that open the active center cleft to allow entry of the nucleic acid scaffold during initiation or that close the cleft around the nucleic acid scaffold to enable processive elongation (44–47). Compared with the structure of TEC, the clamp in MTC rotates open by ∼14° and the DNA–RNA hybrid becomes disordered due to the loss of interaction between the hybrid and the active center cleft (Figure 5A).

Can Mfd bind to the clamp and the upstream dsDNA in the same way as in MTC if the clamp is closed? To answer this question, the structure of TEC was used as a reference to superimpose the structures of MTC via α-carbon atoms of the clamp, revealing severe clashes between the upstream dsDNA and the RNAP β1 domain (Figure 5B). Therefore, Mfd would not be able to bind to the clamp and the upstream dsDNA in the same way as in MTC if the clamp is closed.

## DISCUSSION

**Figure 6.**
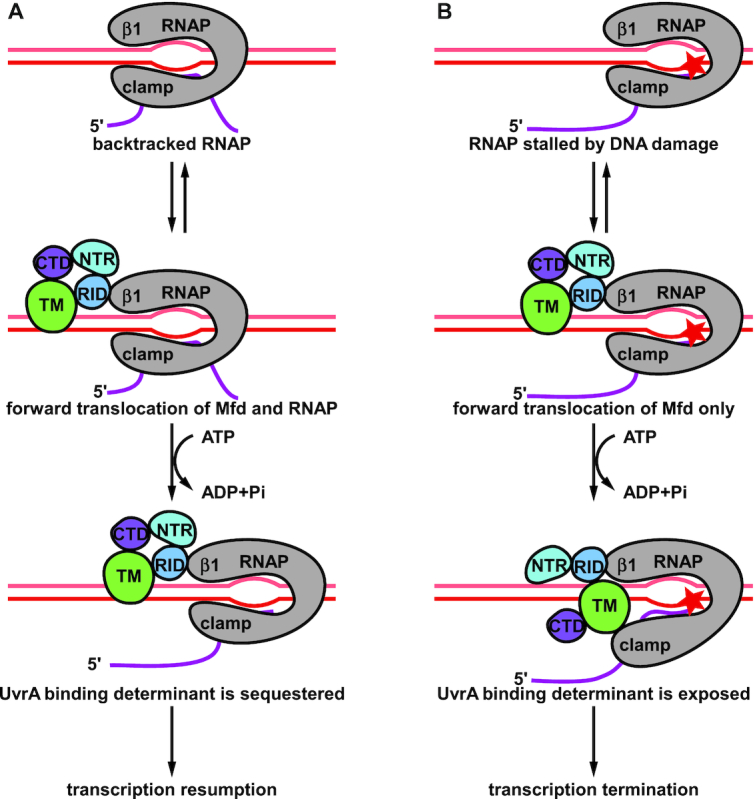
Proposed model of Mfd-dependent rescue and Mfd-dependent transcription termination. (**A**) If Mfd encounters a backtracked RNAP, the RID binds to the RNAP and the TM binds to the upstream dsDNA. Mfd and RNAP translocate forward together via ATP hydrolysis. When the 3′ end of the RNA aligns with the active center, transcription resumes as long as NTPs are available. (**B**) If Mfd encounters an RNAP stalled by DNA damage, the RID binds to the RNAP and the TM binds to the upstream dsDNA. The TM translocates forward via ATP hydrolysis, but the RNAP and the RID stay stationary due to the DNA damage. Eventually, the TM steps into and pries open the clamp, which will facilitate the dissociation of DNA-RNA hybrid. During this process, the UvrA binding determinant of Mfd gets exposed.

In this work, we determined the cryo-EM structures of Mfd-dependent transcription termination complex with and without ATPγS, revealing the precise mechanism of Mfd activation and translocation. Dots are connected based on this work and previous studies (Figure 6). In the absence of DNA, the two helices of the TRG motif adopt the perpendicular configuration, and the UvrA binding determinant is sequestered by the CTD (11,17,18). After binding to DNA, the two helices of the TRG motif turn into the antiparallel configuration and couple ATP hydrolysis to duplex translocation (11,39).

If Mfd encounters a backtracked RNAP, the RID binds to the RNAP β1 domain (Figure 6A). In the meantime, Mfd translocates on the upstream dsDNA and pushes RNAP forward via ATP hydrolysis. As soon as the 3′ end of the RNA aligns with the active center, transcription resumes. Because the rate of transcription elongation (∼14 bp/s) is faster than the rate of Mfd translocation (∼7 bp/s) (40), RNAP moves forward and leaves Mfd behind. During this process, the UvrA binding determinant of Mfd remains sequestered by the CTD, so the NER machinery will not be recruited.

If Mfd encounters an RNAP stalled by DNA damage, the RID binds to the RNAP β1 domain (Figure 6B). In the meantime, the TM translocates on the upstream dsDNA via ATP hydrolysis. However, RNAP cannot move forward due to the DNA damage. The RID cannot move forward either, due to its interaction with the RNAP. Therefore, the RID translates a long distance relative to the TM after several cycles of ATP hydrolysis. When the TM steps into the clamp of RNAP, it pushes against and pries open the clamp. During this process, ATP hydrolysis drives profound conformational changes of Mfd, including the translation of the RID and the exposure of the UvrA binding determinant. Therefore, although Mfd is capable of binding both paused RNAP and RNAP stalled by DNA damage, only in the case of DNA damage can Mfd complete the conformational change to expose the UvrA binding determinant and recruit NER machinery. This model is consistent with the observation that ATP hydrolysis is required for Mfd activation (15). This model is also consistent with the proposal that Mfd kinetically discriminates stalled RNAP from backtracked RNAP (15,40,48).

A closed clamp is critical for the processivity of transcription elongation. Even when RNAP is paused by backtracking or an RNA hairpin in the RNA exit channel, the clamp remains closed (49–51). On the contrary, the clamp is open in MTC structures. The open clamp loses its interaction with the DNA-RNA hybrid and probably aids the dissociation of the DNA–RNA hybrid.

Rad26, a Swi2/Snf2 family helicase, is among the first proteins to be recruited to Pol II during the initiation of *S. cerevisiae* TCR. The cryo-EM structure of Rad26 in complex with Pol II shows that Rad26 binds upstream of Pol II and translocates toward Pol II, suggesting Rad26 may play a role similar to that of Mfd (52). However, structural analysis reveals the divergences between Rad26 and Mfd (Supplementary Figure S12). First, the clamp is closed and the transcription bubble is ordered in Rad26-Pol II complex, while Mfd pries open the clamp and disrupts the contacts between the transcription bubble and RNAP. Second, Rad26 bends the upstream dsDNA by ∼80°, while Mfd has a less dramatic impact on the conformation of the upstream dsDNA. Third, the second-largest subunit of Pol II is the major contact site of Rad26, while Mfd contacts both the largest and the second-largest subunits of the polymerase.

Besides its role in TCR, Mfd is proposed to be an ‘evolvability factor’ that promotes mutagenesis and is required for rapid resistance development to antibiotics (8,9). Therefore, Mfd may be an ideal target for ‘anti-evolution’ drugs that inhibit antimicrobial resistance development. The structures of Mfd in action provide a basis for rational drug design targeting Mfd. For example, because the conformational change of the TRG motif is critical for the function of Mfd, inhibitors might be designed to lock the conformation of the TRG motif.

## DATA AVAILABILITY

The accession numbers for the cryo-EM density map reported in this paper is Electron Microscopy Data Bank: EMD-30117 (MTC^apo^), EMD-30118 (MTC^ATPγS^), and EMD-30119 (TEC). The accession numbers for the atomic coordinates reported in this paper are Protein Data Bank: 6M6A (MTC^apo^), 6M6B (MTC^ATPγS^), and 6M6C (TEC).

## Supplementary Material

gkaa904_Supplemental_Files

## References

[1] Marteijn J.A., Lans H., Vermeulen W., Hoeijmakers J.H. Understanding nucleotide excision repair and its roles in cancer and ageing. Nat. Rev. Mol. Cell Biol. 2014; 15:465–481. 24954209 10.1038/nrm3822

[2] Hanawalt P.C., Spivak G. Transcription-coupled DNA repair: two decades of progress and surprises. Nat. Rev. Mol. Cell Biol. 2008; 9:958–970. 19023283 10.1038/nrm2549

[3] Selby C.P., Sancar A. Molecular mechanism of transcription-repair coupling. Science. 1993; 260:53–58. 8465200 10.1126/science.8465200

[4] Adebali O., Chiou Y.Y., Hu J., Sancar A., Selby C.P. Genome-wide transcription-coupled repair in *Escherichia coli* is mediated by the Mfd translocase. Proc. Natl. Acad. Sci. USA. 2017; 114:E2116–E2125. 28167766 10.1073/pnas.1700230114 PMC5358382

[5] Hamperl S., Cimprich K.A. Conflict resolution in the genome: how transcription and replication make it work. Cell. 2016; 167:1455–1467. 27912056 10.1016/j.cell.2016.09.053 PMC5141617

[6] Trautinger B.W., Jaktaji R.P., Rusakova E., Lloyd R.G. RNA polymerase modulators and DNA repair activities resolve conflicts between DNA replication and transcription. Mol. Cell. 2005; 19:247–258. 16039593 10.1016/j.molcel.2005.06.004

[7] Dutta D., Shatalin K., Epshtein V., Gottesman M.E., Nudler E. Linking RNA polymerase backtracking to genome instability in *E. coli*. Cell. 2011; 146:533–543. 21854980 10.1016/j.cell.2011.07.034 PMC3160732

[8] Han J., Sahin O., Barton Y.W., Zhang Q. Key role of Mfd in the development of fluoroquinolone resistance in *Campylobacter jejuni*. PLoS Pathog. 2008; 4:e1000083. 18535657 10.1371/journal.ppat.1000083 PMC2390758

[9] Ragheb M.N., Thomason M.K., Hsu C., Nugent P., Gage J., Samadpour A.N., Kariisa A., Merrikh C.N., Miller S.I., Sherman D.R. et al. Inhibiting the evolution of antibiotic resistance. Mol. Cell. 2019; 73:157–165. 30449724 10.1016/j.molcel.2018.10.015 PMC6320318

[10] Park J.S., Marr M.T., Roberts J.W. *E. coli* Transcription repair coupling factor (Mfd protein) rescues arrested complexes by promoting forward translocation. Cell. 2002; 109:757–767. 12086674 10.1016/s0092-8674(02)00769-9

[11] Deaconescu A.M., Chambers A.L., Smith A.J., Nickels B.E., Hochschild A., Savery N.J., Darst S.A. Structural basis for bacterial transcription-coupled DNA repair. Cell. 2006; 124:507–520. 16469698 10.1016/j.cell.2005.11.045

[12] Smith A.J., Savery N.J. RNA polymerase mutants defective in the initiation of transcription-coupled DNA repair. Nucleic Acids Res. 2005; 33:755–764. 15687384 10.1093/nar/gki225 PMC548365

[13] Westblade L.F., Campbell E.A., Pukhrambam C., Padovan J.C., Nickels B.E., Lamour V., Darst S.A. Structural basis for the bacterial transcription-repair coupling factor/RNA polymerase interaction. Nucleic Acids Res. 2010; 38:8357–8369. 20702425 10.1093/nar/gkq692 PMC3001067

[14] Selby C.P., Sancar A. Structure and function of transcription-repair coupling factor. I. Structural domains and binding properties. J. Biol. Chem. 1995; 270:4882–4889. 7876261 10.1074/jbc.270.9.4882

[15] Ho Wan K., Smith A.J., Westblade L.F., Joly N., Grange W., Zorman S., Darst S.A., Savery N.J., Strick T.R. Initiation of transcription-coupled repair characterized at single-molecule resolution. Nature. 2012; 490:431–434. 22960746 10.1038/nature11430 PMC3475728

[16] Chambers A.L., Smith A.J., Savery N.J. A DNA translocation motif in the bacterial transcription–repair coupling factor, Mfd. Nucleic Acids Res. 2003; 31:6409–6418. 14602898 10.1093/nar/gkg868 PMC275562

[17] Deaconescu A.M., Sevostyanova A., Artsimovitch I., Grigorieff N. Nucleotide excision repair (NER) machinery recruitment by the transcription-repair coupling factor involves unmasking of a conserved intramolecular interface. Proc. Natl. Acad. Sci. U.S.A. 2012; 109:3353–3358. 22331906 10.1073/pnas.1115105109 PMC3295266

[18] Manelyte L., Kim Y.I., Smith A.J., Smith R.M., Savery N.J. Regulation and rate enhancement during transcription-coupled DNA repair. Mol. Cell. 2010; 40:714–724. 21145481 10.1016/j.molcel.2010.11.012 PMC3025350

[19] Murphy M.N., Gong P., Ralto K., Manelyte L., Savery N.J., Theis K. An N-terminal clamp restrains the motor domains of the bacterial transcription-repair coupling factor Mfd. Nucleic Acids Res. 2009; 37:6042–6053. 19700770 10.1093/nar/gkp680 PMC2764443

[20] Park J.S., Roberts J.W. Role of DNA bubble rewinding in enzymatic transcription termination. Proc. Natl. Acad. Sci. U.S.A. 2006; 103:4870–4875. 16551743 10.1073/pnas.0600145103 PMC1405909

[21] Smith A.J., Szczelkun M.D., Savery N.J. Controlling the motor activity of a transcription-repair coupling factor: autoinhibition and the role of RNA polymerase. Nucleic Acids Res. 2007; 35:1802–1811. 17329375 10.1093/nar/gkm019 PMC1874598

[22] Srivastava D.B., Darst S.A. Derepression of bacterial transcription-repair coupling factor is associated with a profound conformational change. J. Mol. Biol. 2011; 406:275–284. 21185303 10.1016/j.jmb.2010.12.004 PMC3031748

[23] Smith A.J., Pernstich C., Savery N.J. Multipartite control of the DNA translocase, Mfd. Nucleic Acids Res. 2012; 40:10408–10416. 22904071 10.1093/nar/gks775 PMC3488230

[24] Shi J., Gao X., Tian T., Yu Z., Gao B., Wen A., You L., Chang S., Zhang X., Zhang Y. et al. Structural basis of Q-dependent transcription antitermination. Nat. Commun. 2019; 10:2925. 31266960 10.1038/s41467-019-10958-8 PMC6606751

[25] Feng Y., Zhang Y., Ebright R.H. Structural basis of transcription activation. Science. 2016; 352:1330–1333. 27284196 10.1126/science.aaf4417 PMC4905602

[26] Mooney R.A., Schweimer K., Rosch P., Gottesman M., Landick R. Two structurally independent domains of *E. coli* NusG create regulatory plasticity *via* distinct interactions with RNA polymerase and regulators. J. Mol. Biol. 2009; 391:341–358. 19500594 10.1016/j.jmb.2009.05.078 PMC2763281

[27] Mastronarde D.N. Automated electron microscope tomography using robust prediction of specimen movements. J. Struct. Biol. 2005; 152:36–51. 16182563 10.1016/j.jsb.2005.07.007

[28] Zheng S.Q., Palovcak E., Armache J.P., Verba K.A., Cheng Y., Agard D.A. MotionCor2: anisotropic correction of beam-induced motion for improved cryo-electron microscopy. Nat. Methods. 2017; 14:331–332. 28250466 10.1038/nmeth.4193 PMC5494038

[29] Rohou A., Grigorieff N. CTFFIND4: fast and accurate defocus estimation from electron micrographs. J. Struct. Biol. 2015; 192:216–221. 26278980 10.1016/j.jsb.2015.08.008 PMC6760662

[30] Scheres S.H. RELION: implementation of a Bayesian approach to cryo-EM structure determination. J. Struct. Biol. 2012; 180:519–530. 23000701 10.1016/j.jsb.2012.09.006 PMC3690530

[31] Kang J.Y., Olinares P.D., Chen J., Campbell E.A., Mustaev A., Chait B.T., Gottesman M.E., Darst S.A. Structural basis of transcription arrest by coliphage HK022 Nun in an *Escherichia coli* RNA polymerase elongation complex. Elife. 2017; 6:e25478. 28318486 10.7554/eLife.25478 PMC5386594

[32] Kelley L.A., Mezulis S., Yates C.M., Wass M.N., Sternberg M.J. The Phyre2 web portal for protein modeling, prediction and analysis. Nat. Protoc. 2015; 10:845–858. 25950237 10.1038/nprot.2015.053 PMC5298202

[33] Zhang Y., Feng Y., Chatterjee S., Tuske S., Ho M.X., Arnold E., Ebright R.H. Structural basis of transcription initiation. Science. 2012; 338:1076–1080. 23086998 10.1126/science.1227786 PMC3593053

[34] Pettersen E.F., Goddard T.D., Huang C.C., Couch G.S., Greenblatt D.M., Meng E.C., Ferrin T.E. UCSF Chimera–a visualization system for exploratory research and analysis. J. Comput. Chem. 2004; 25:1605–1612. 15264254 10.1002/jcc.20084

[35] Emsley P., Cowtan K. Coot: model-building tools for molecular graphics. Acta Crystallogr. D. 2004; 60:2126–2132. 15572765 10.1107/S0907444904019158

[36] Adams P.D., Afonine P.V., Bunkoczi G., Chen V.B., Davis I.W., Echols N., Headd J.J., Hung L.W., Kapral G.J., Grosse-Kunstleve R.W. et al. PHENIX: a comprehensive Python-based system for macromolecular structure solution. Acta Crystallogr. D. 2010; 66:213–221. 20124702 10.1107/S0907444909052925 PMC2815670

[37] Mahdi A.A., Briggs G.S., Sharples G.J., Wen Q., Lloyd R.G. A model for dsDNA translocation revealed by a structural motif common to RecG and Mfd proteins. EMBO J. 2003; 22:724–734. 12554672 10.1093/emboj/cdg043 PMC140728

[38] Assenmacher N., Wenig K., Lammens A., Hopfner K.P. Structural basis for transcription-coupled repair: the N terminus of Mfd resembles UvrB with degenerate ATPase motifs. J. Mol. Biol. 2006; 355:675–683. 16309703 10.1016/j.jmb.2005.10.033

[39] Brugger C., Zhang C., Suhanovsky M.M., Kim D.D., Sinclair A.N., Lyumkis D., Deaconescu A.M. Molecular determinants for dsDNA translocation by the transcription-repair coupling and evolvability factor Mfd. Nat. Commun. 2020; 11:3740. 32719356 10.1038/s41467-020-17457-1 PMC7385628

[40] Le T.T., Yang Y., Tan C., Suhanovsky M.M., Fulbright R.M. Jr, Inman J.T., Li M., Lee J., Perelman S., Roberts J.W. et al. Mfd dynamically regulates transcription via a release and catch-up mechanism. Cell. 2018; 173:344–357. 10.1016/j.cell.2017.11.017 PMC5766421 29224782

[41] Kang J.Y., Mooney R.A., Nedialkov Y., Saba J., Mishanina T.V., Artsimovitch I., Landick R., Darst S.A. Structural basis for transcript elongation control by NusG family universal regulators. Cell. 2018; 173:1650–1662. 29887376 10.1016/j.cell.2018.05.017 PMC6003885

[42] Mooney R.A., Davis S.E., Peters J.M., Rowland J.L., Ansari A.Z., Landick R. Regulator trafficking on bacterial transcription units *in vivo. Mol*. Cell. 2009; 33:97–108. 10.1016/j.molcel.2008.12.021 PMC2747249 19150431

[43] Zhang G., Campbell E.A., Minakhin L., Richter C., Severinov K., Darst S.A. Crystal structure of Thermus aquaticus core RNA polymerase at 3.3 A resolution. Cell. 1999; 98:811–824. 10499798 10.1016/s0092-8674(00)81515-9

[44] Gnatt A.L., Cramer P., Fu J., Bushnell D.A., Kornberg R.D. Structural basis of transcription: an RNA polymerase II elongation complex at 3.3 A resolution. Science. 2001; 292:1876–1882. 11313499 10.1126/science.1059495

[45] Chakraborty A., Wang D., Ebright Y.W., Korlann Y., Kortkhonjia E., Kim T., Chowdhury S., Wigneshweraraj S., Irschik H., Jansen R. et al. Opening and closing of the bacterial RNA polymerase clamp. Science. 2012; 337:591–595. 22859489 10.1126/science.1218716 PMC3626110

[46] Feklistov A., Bae B., Hauver J., Lass-Napiorkowska A., Kalesse M., Glaus F., Altmann K.H., Heyduk T., Landick R., Darst S.A. RNA polymerase motions during promoter melting. Science. 2017; 356:863–866. 28546214 10.1126/science.aam7858 PMC5696265

[47] Boyaci H., Chen J., Jansen R., Darst S.A., Campbell E.A. Structures of an RNA polymerase promoter melting intermediate elucidate DNA unwinding. Nature. 2019; 565:382–385. 30626968 10.1038/s41586-018-0840-5 PMC6399747

[48] Fan J., Leroux-Coyau M., Savery N.J., Strick T.R. Reconstruction of bacterial transcription-coupled repair at single-molecule resolution. Nature. 2016; 536:234–237. 27487215 10.1038/nature19080

[49] Abdelkareem M., Saint-Andre C., Takacs M., Papai G., Crucifix C., Guo X., Ortiz J., Weixlbaumer A. Structural basis of transcription: RNA polymerase backtracking and its reactivation. Mol. Cell. 2019; 75:298–309. 31103420 10.1016/j.molcel.2019.04.029 PMC7611809

[50] Kang J.Y., Mishanina T.V., Bellecourt M.J., Mooney R.A., Darst S.A., Landick R. RNA polymerase accommodates a pause RNA hairpin by global conformational rearrangements that prolong pausing. Mol. Cell. 2018; 69:802–815. 29499135 10.1016/j.molcel.2018.01.018 PMC5903582

[51] Guo X., Myasnikov A.G., Chen J., Crucifix C., Papai G., Takacs M., Schultz P., Weixlbaumer A. Structural basis for NusA stabilized transcriptional pausing. Mol. Cell. 2018; 69:816–827. 29499136 10.1016/j.molcel.2018.02.008 PMC5842316

[52] Xu J., Lahiri I., Wang W., Wier A., Cianfrocco M.A., Chong J., Hare A.A., Dervan P.B., DiMaio F., Leschziner A.E. et al. Structural basis for the initiation of eukaryotic transcription-coupled DNA repair. Nature. 2017; 551:653–657. 29168508 10.1038/nature24658 PMC5907806

