# Supplementary material for: Structural basis of Mfd-dependent transcription termination
Source: Nucleic Acids Res. 2020 Oct 17;48(20):11762–72. doi: 10.1093/nar/gkaa904 (PMC7672476; doi:10.1093/nar/gkaa904)
Supplement: gkaa904_Supplemental_Files [file gkaa904_supplemental_files.zip › Supplementary Information 09042020.pdf]

Structural basis of Mfd-dependent transcription termination

Shi et al.

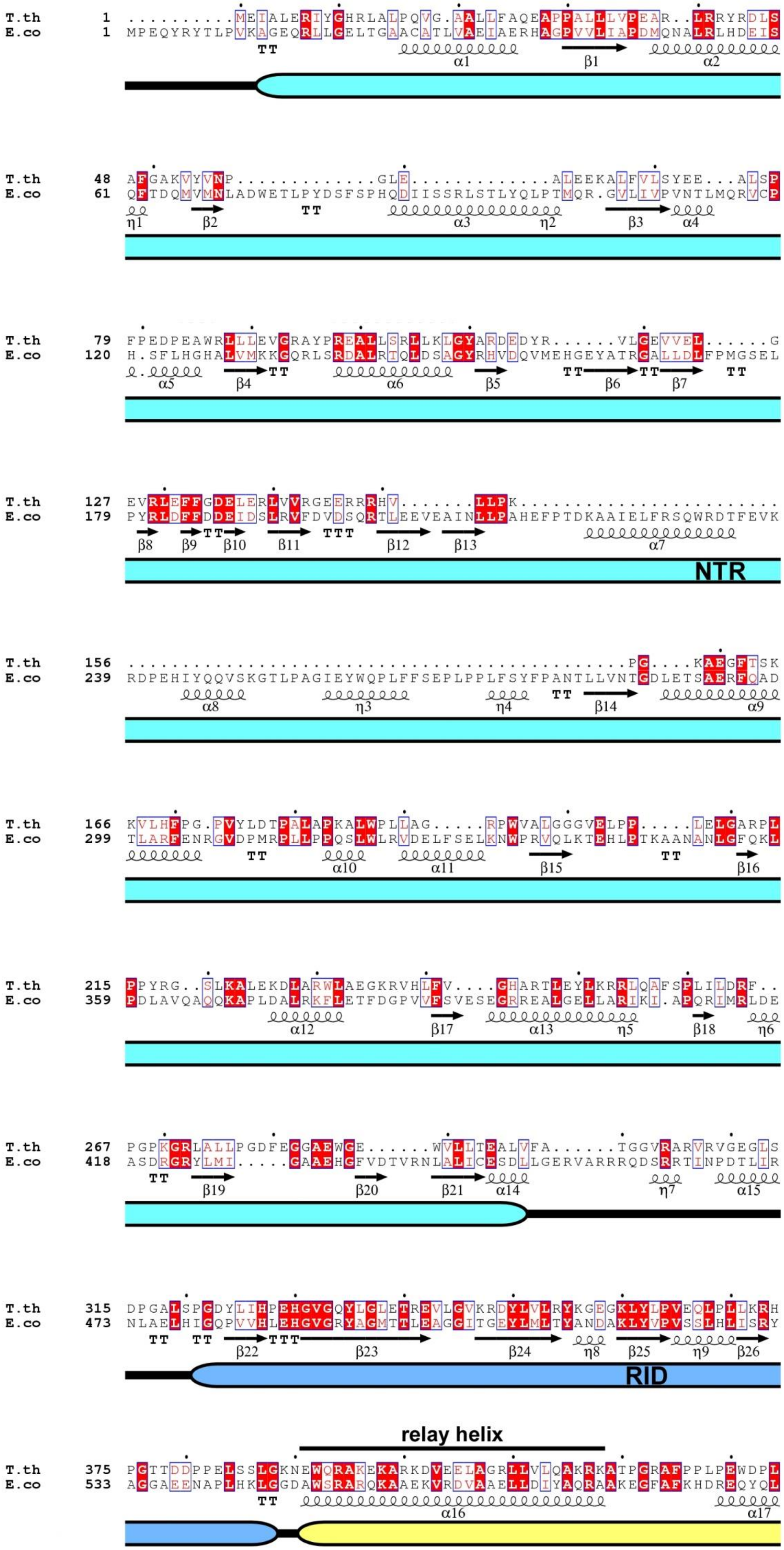
T.th
E.co
NTR
RID
relay helix

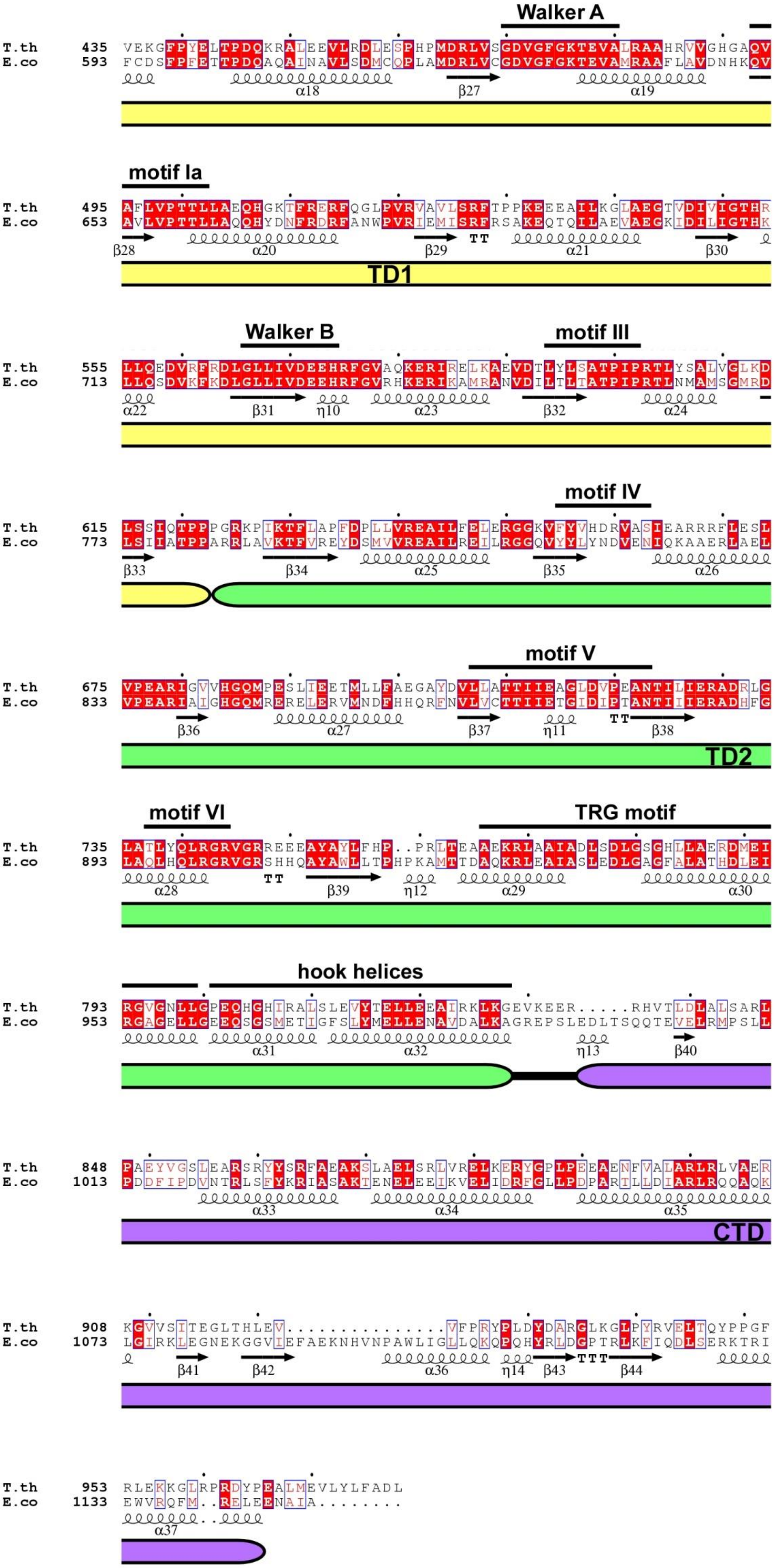
Walker A
T.th 435 VEKGFPYELTPDQKRALEEVLRDLESPHPMDRLVSGDVGFGKTEVALRAAHRVVGHGAQV
E.co 593 FCDSFPFETTPDQAQAINAVLSDMCQPLAMDRLVCGDVGFGKTEVAMRAAFLAVDNHKQV
α18 β27 α19
motif Ia
T.th 495 AFLVPTTLLAEQHGKTFRERFQGLPVRVAVLSRFTPPKEEEAILKGLAEGTVDIVIGTHR
E.co 653 AVLVPTTLLAQQHYDNFRDRFANWPVRIEMISRFRSAKEQTQILAEVAEGKIDILIGTHK
β28 α20 β29 TT α21 β30
TD1
Walker B
motif III
T.th 555 LLQEDVRFRDLGLLIVDEEHRFGVAQKERIRELKAEVDTLYLSATPIPRTLYSALVGLKD
E.co 713 LLQSDVKFKDLGLLIVDEEHRFGVRHKERIKAMRANVDILTLTATPIPRTLNMAMSGMRD
α22 β31 η10 α23 β32 α24
motif IV
T.th 615 LSSIQTPPPGRKPIKTFLAPFDPLLVREAILFELERGGKVFYVHDRVASIEARRRFLESL
E.co 773 LSIIATPPARRLAVKTFVREYDSMVVREAILREILRGGQVYYLYNDVENIQKAAERLAEL
β33 β34 α25 β35 α26
motif V
T.th 675 VPEARIGVVHGQMPESLIEETMLLFAEGAYDVLLATTIIEAGLDVPEANTLIIERADRLG
E.co 833 VPEARIAIGHGQMRERELERVMNDFHHQRFNVLVCTTIIETGIDIPTANTIIIERADHFG
β36 α27 β37 η11 TT β38
TD2
motif VI
TRG motif
T.th 735 LATLYQLRGRVGRREEEAYAYLFHP..PRLTEAAEKRLAAIADLSDLGSGHLLAERDMEI
E.co 893 LAQLHQLRGRVGRSHHQAYAWLLTPHPKAMTTDAQKRLEAIASLEDLGAGFALATHDLEI
α28 TT β39 η12 α29 α30
hook helices
T.th 793 RGVGNLLGPEQHGHIRALSLEVYTELLEEAIRKLKGEVKEER.....RHVTLDLALSARL
E.co 953 RGAGELLGEEQSGSMETIGFSLYMELLENAVDALKAGREPSLEDLTSQQTEVELRMPSLL
α31 α32 η13 β40
T.th 848 PAEYVGSLEARSRYYSRFAEAKSLAELSRLVRELKERYGPLPEEAENFVALARLRLVAER
E.co 1013 PDDFIPDVNTRLSFYKRIASAKTENELEEIKVELIDRFGLLPDPARTLLDIARLRQQAQK
α33 α34 α35
CTD
T.th 908 KGVVSITEGLTHLEV...............VFPRYPLDYDARGLKGLPYRVELTQYPPGF
E.co 1073 LGIRKLEGNEKGGVIEFAEKNHVNPAWLIGLLQKQPQHYRLDGPTRLKFIQDLSERKTRI
β41 β42 α36 η14 β43 TTT β44
T.th 953 RLEKKGLRPRDYPEALMEVLYLFADL
E.co 1133 EWVRQFM..RELEENAIA........
α37

Figure S1. Sequence alignment of *T. thermophilus* Mfd and *E. coli* Mfd.

The sequences were aligned using Clustal Omega and the figure was prepared using ESPript 3.0 (1).

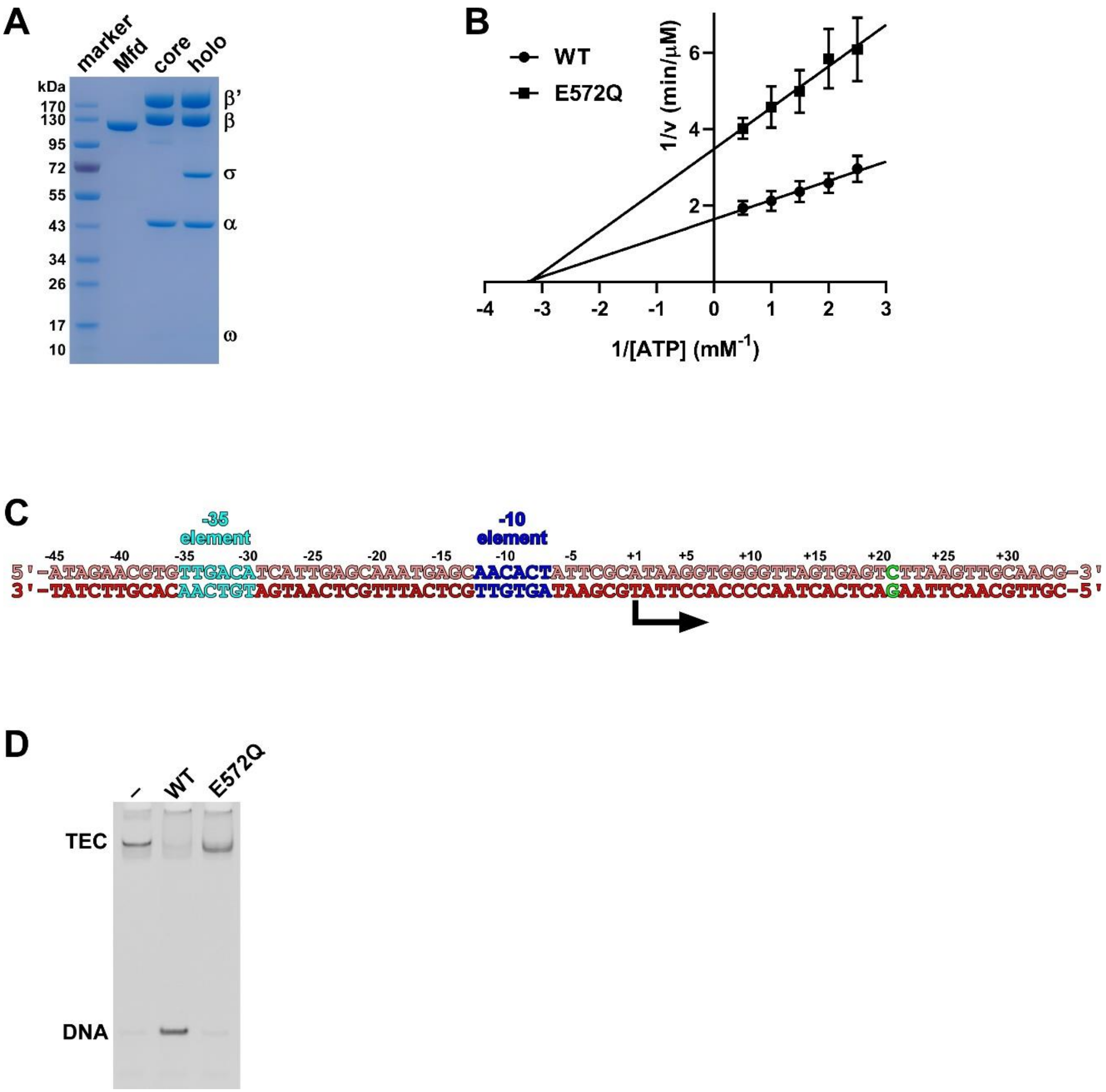

Figure S2. *T. thermophilus* Mfd displaces RNAP stalled by NTP starvation.

(A) SDS-PAGE of *T. thermophilus* Mfd and RNAP.

(B) ATP hydrolysis activity of *T. thermophilus* Mfd. *T. thermophilus* Mfd hydrolyzes ATP, while substitution of a conserved Walker B residue E572 affects ATP hydrolysis, but not ATP binding. WT: k_{cat}=0.122 min^{-1}, K_m=0.306 mM; E572Q: k_{cat}=0.057 min^{-1}, K_m=0.311 mM.

(C) Nucleic acid scaffold sequence used for RNAP displacement assay. The sequence was modified from bacteriophage 21 late gene promoter, with the first transcribed C:G base pair at position +21. Salmon, nontemplate strand DNA; red, template strand DNA. Positions are numbered relative to the transcription start site.

(D) *T. thermophilus* Mfd displaces RNAP stalled by NTP starvation, while substitution of residue E572, which is deficient in ATP hydrolysis, failed to displace stalled RNAP.

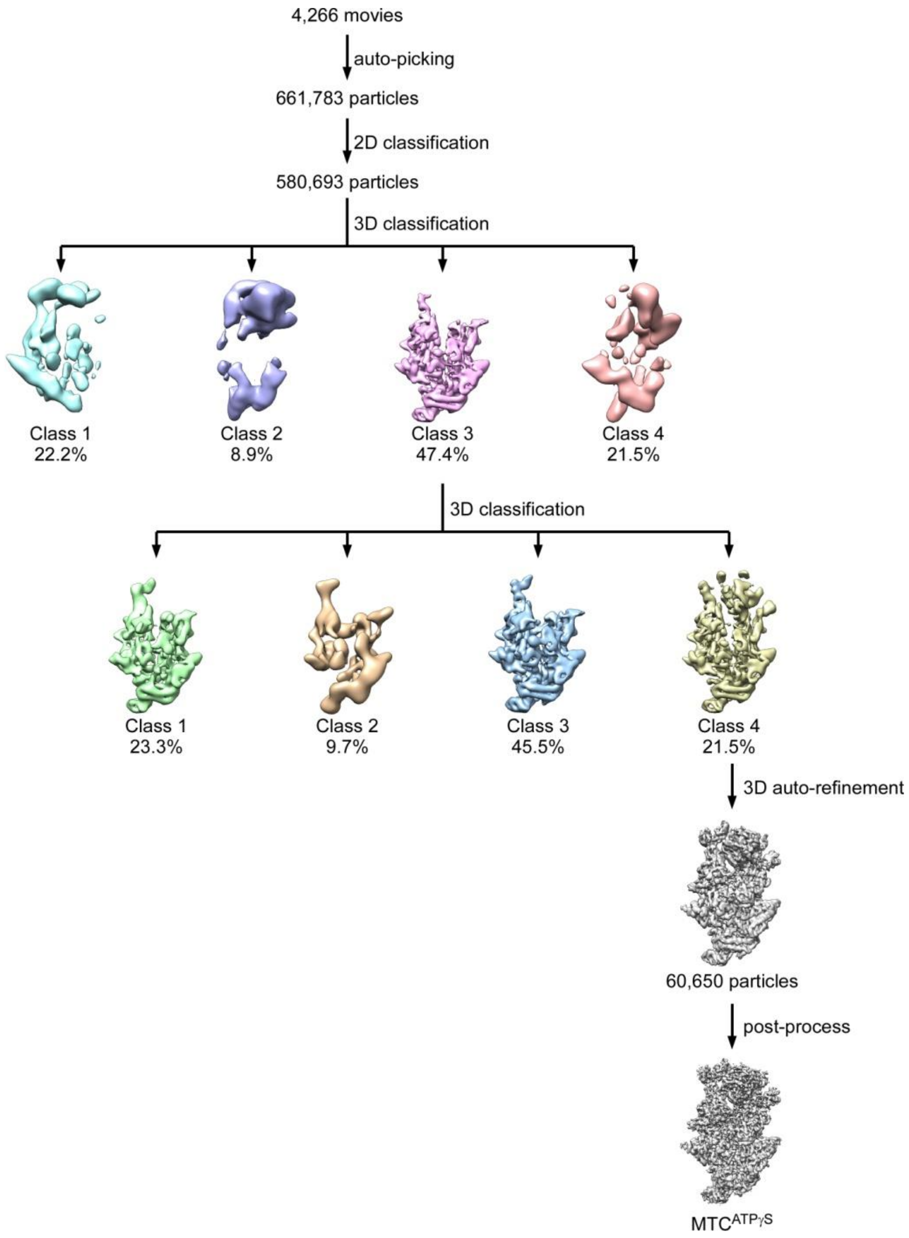

Figure S3. Data processing pipeline for the dataset of MTC$^{ATP\gamma S}$.

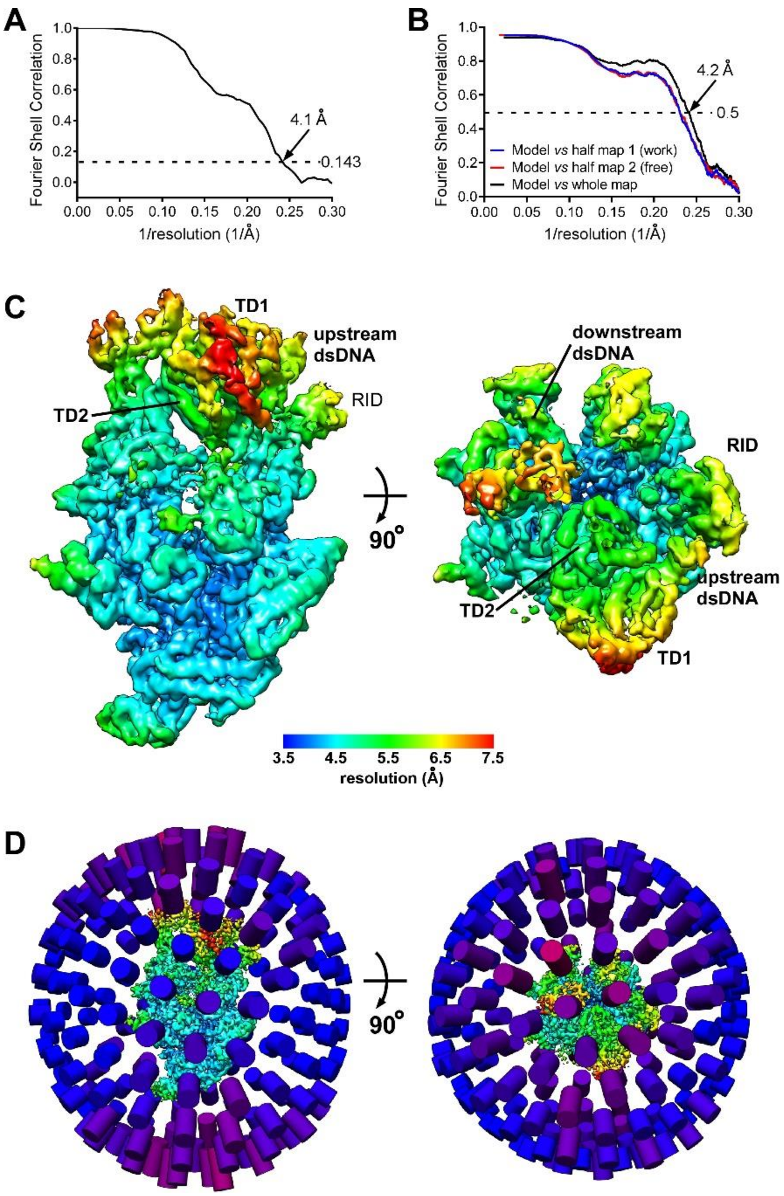

Figure S4. Data validation for MTC$^{ATP\gamma S}$.

(A) Gold-standard FSC. The gold-standard FSC was calculated by comparing the two independently determined half-maps from RELION. The dashed line represents the 0.143 FSC cutoff.

(B) FSC calculated between the model and the half map used for refinement (work), the other half map (free), and the full map.

(C) Cryo-EM density map colored by local resolution. Local resolution calculation was performed using blocres (2). View orientations as in Figure 1C.

(D) Angular distribution of particle projections. View orientations as in (C).

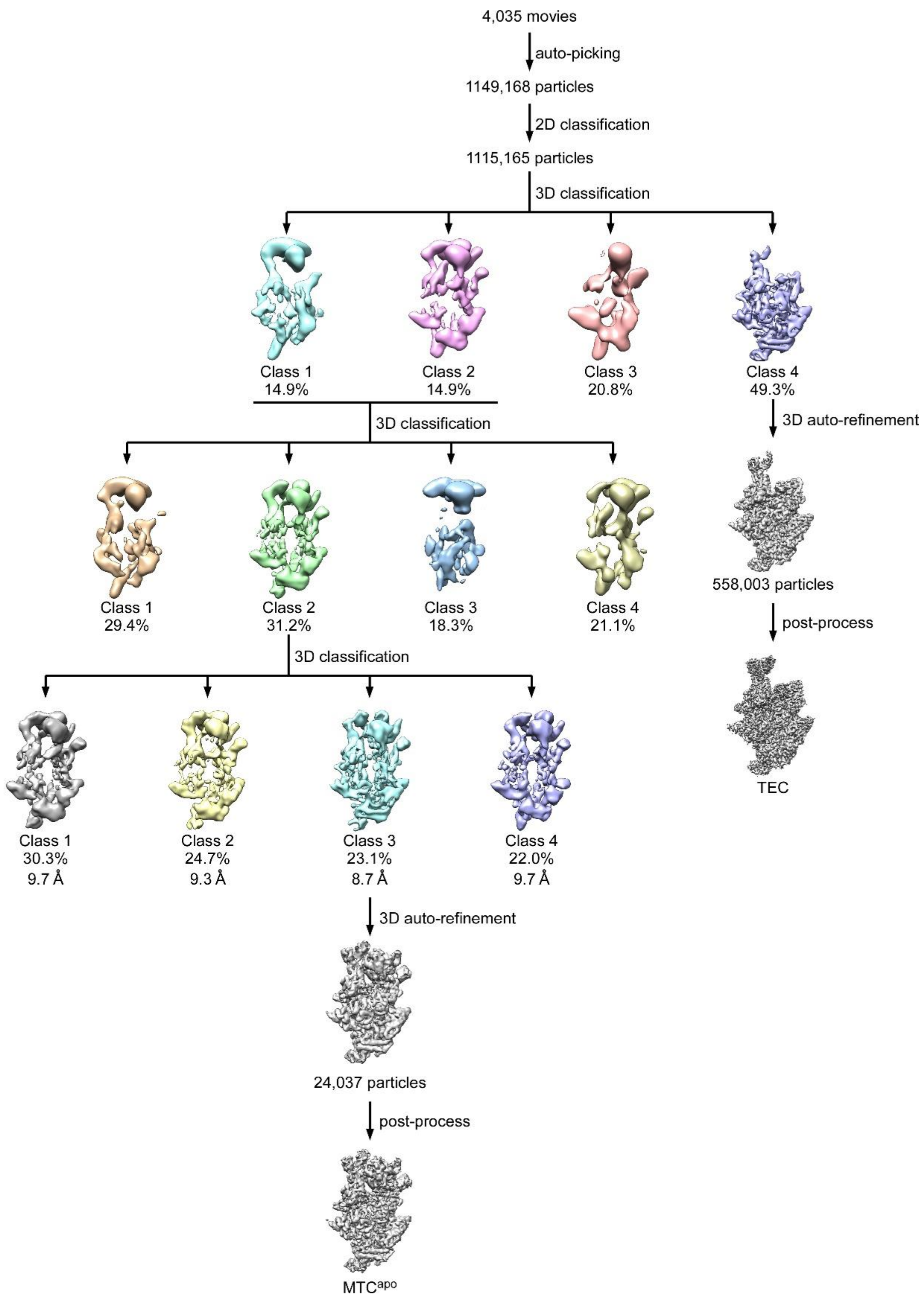

Figure S5. Data processing pipeline for the dataset of MTC^{apo} and TEC.

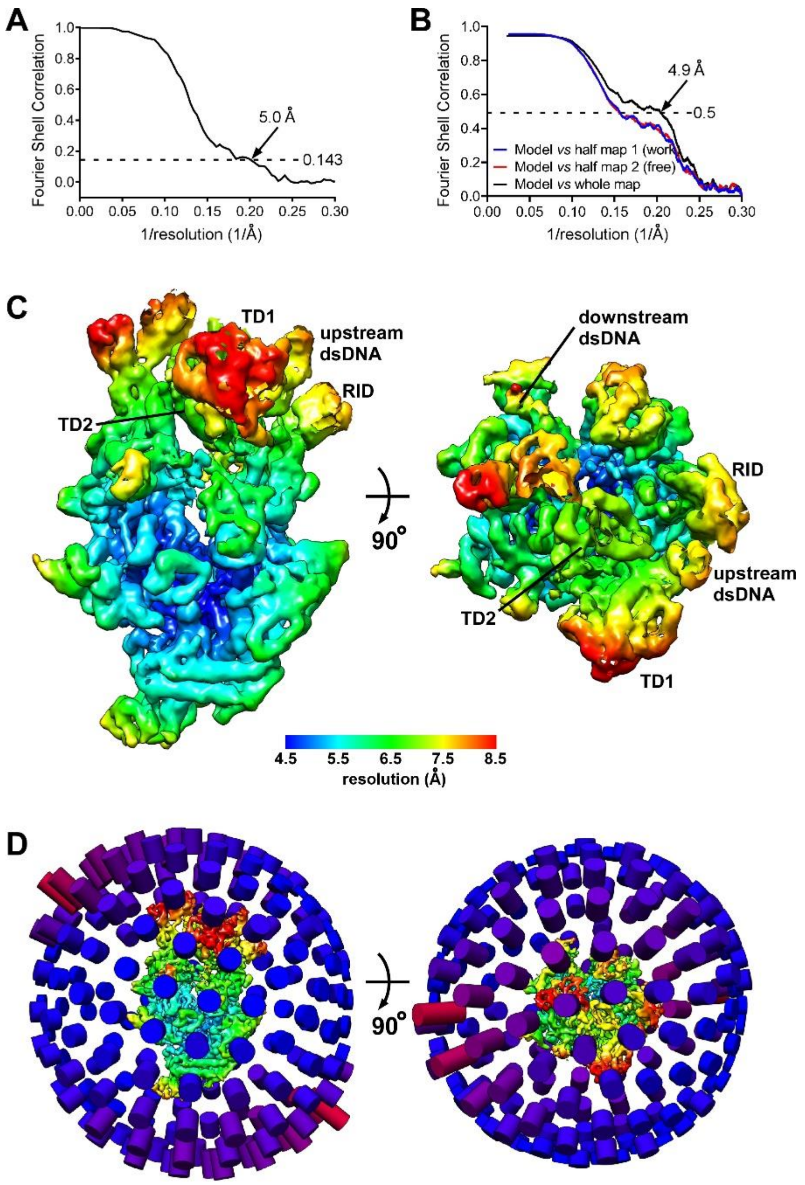

Figure S6. Data validation for MTC^{apo}.

(A) Gold-standard FSC. The gold-standard FSC was calculated by comparing the two independently determined half-maps from RELION. The dashed line represents the 0.143 FSC cutoff.

(B) FSC calculated between the model and the half map used for refinement (work), the other half map (free), and the full map.

(C) Cryo-EM density map colored by local resolution. Local resolution calculation was performed using blocres (2). View orientations as in Figure 1C.

(D) Angular distribution of particle projections. View orientations as in (C).

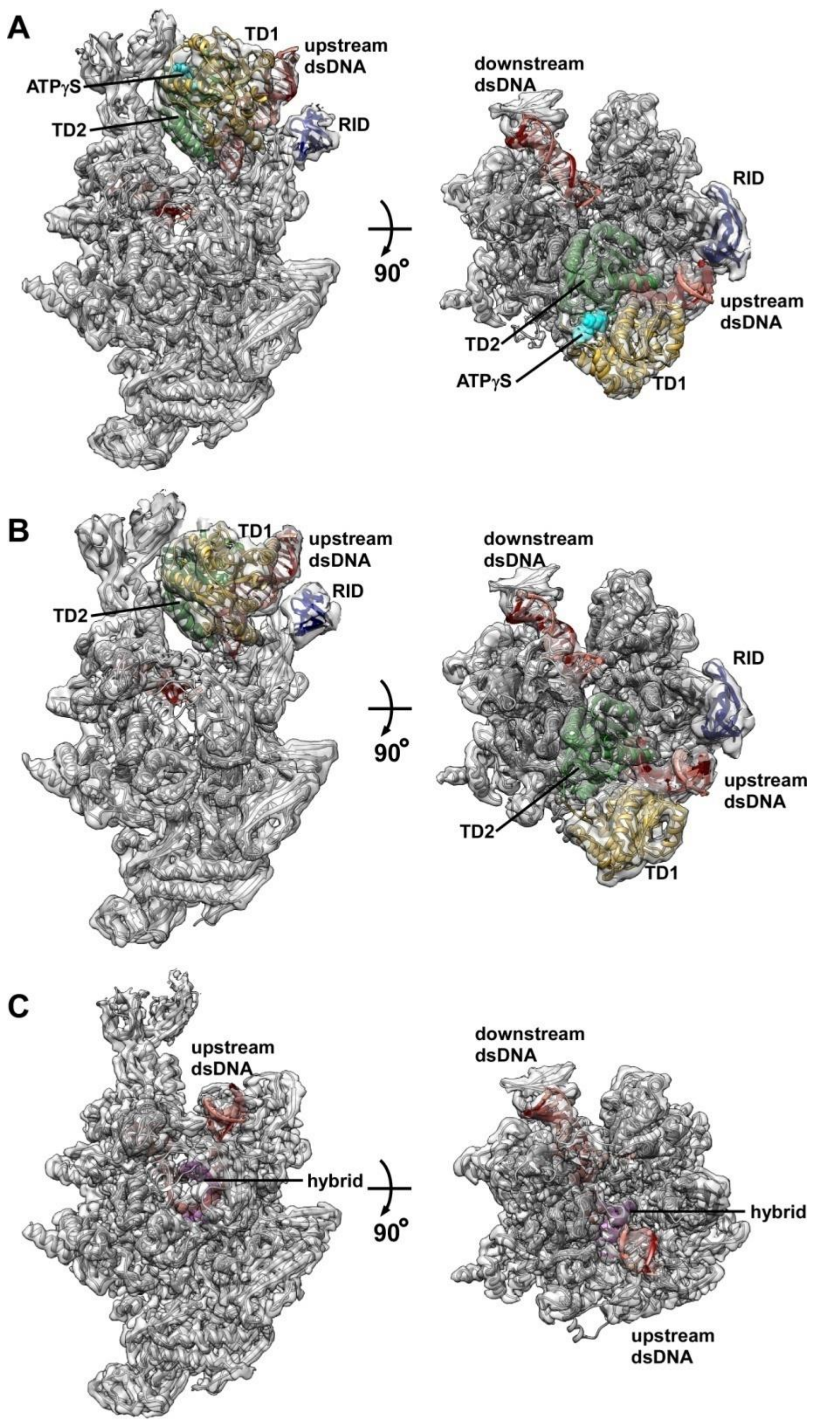

Figure S7. Overall cryo-EM densities and superimposed models.

(A) The cryo-EM density map without B-factor sharpening and the superimposed model of $MTC^{ATP\gamma S}$. View orientation and colors as in Figure 1C.

(B) The cryo-EM density map without B-factor sharpening and the superimposed model of MTC^{apo}. View orientation and colors as in Figure 1C.

(C) The cryo-EM density map without B-factor sharpening and the superimposed model of TEC. View orientation and colors as in Figure 1C.

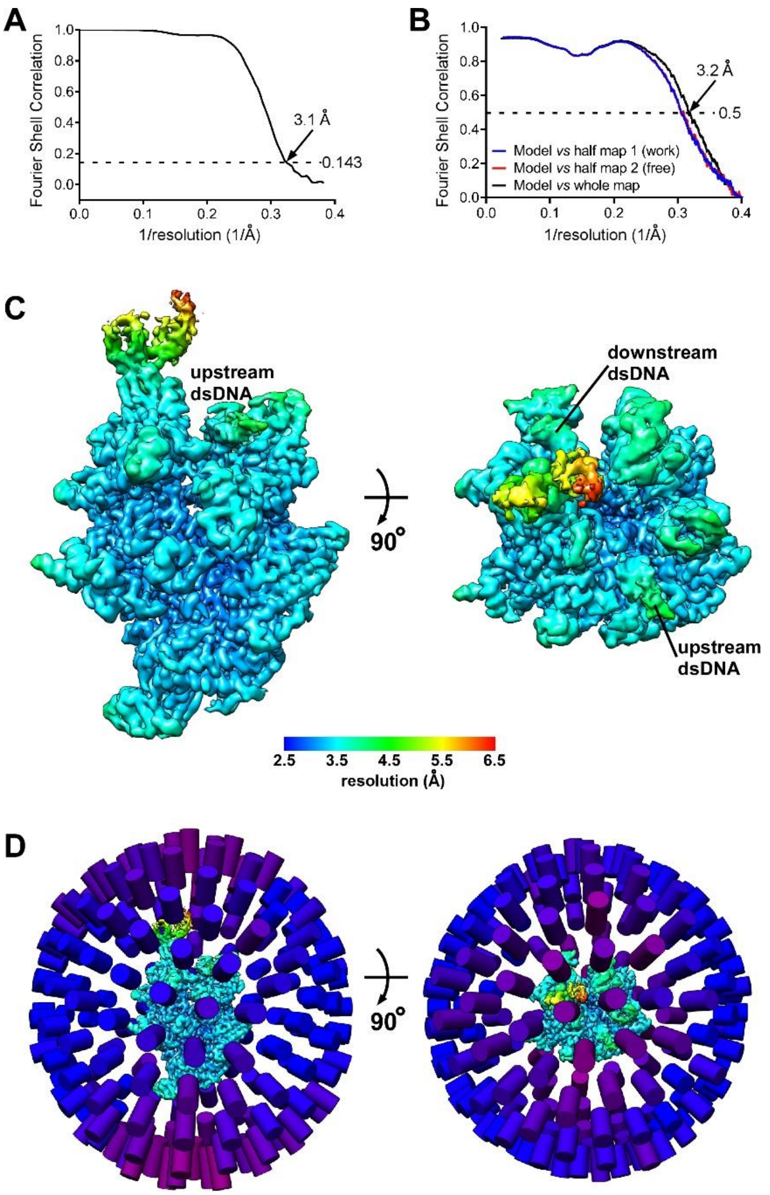

Figure S8. Data validation for TEC.

(A) Gold-standard FSC. The gold-standard FSC was calculated by comparing the two independently determined half-maps from RELION. The dashed line represents the 0.143 FSC cutoff.

(B) FSC calculated between the model and the half map used for refinement (work), the other half map (free), and the full map.

(C) Cryo-EM density map colored by local resolution. Local resolution calculation was performed using blocres (2). View orientations as in Figure 1C.

(D) Angular distribution of particle projections. View orientations as in (C).

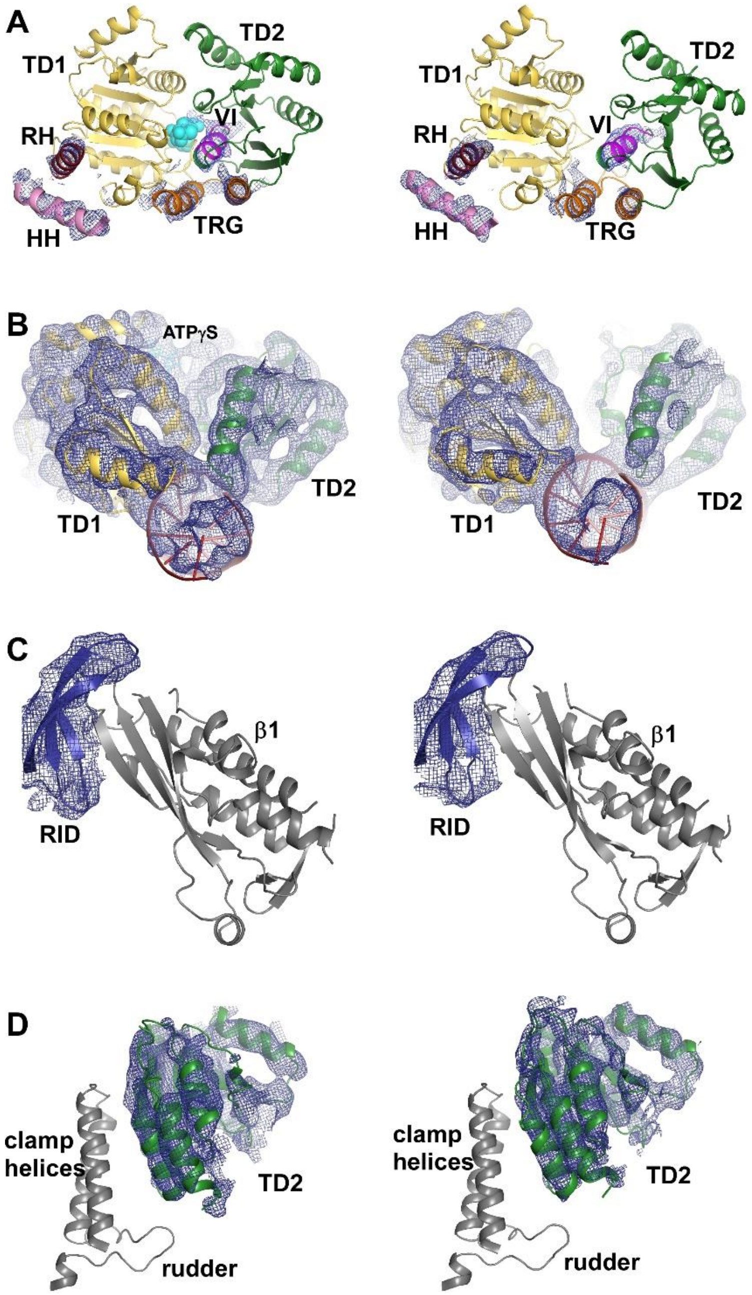

Figure S9. Representative cryo-EM densities and superimposed models.

(A) Cryo-EM density map (blue mesh) without B-factor sharpening and the superimposed model of TRG, RH, and HH. Left, $MTC^{ATP\gamma S}$; right, MTC^{apo}. View orientation and colors as in Figure 2.

(B) Cryo-EM density map (blue mesh) without B-factor sharpening and the superimposed model of TM and upstream dsDNA. Left, $MTC^{ATP\gamma S}$; right, MTC^{apo}. View orientation and colors as in the left subpanel of Figure 3E.

(C) Cryo-EM density map (blue mesh) without B-factor sharpening and the superimposed model of RID. Left, $MTC^{ATP\gamma S}$; right, MTC^{apo}. View orientation and colors as in Figure 4A.

(D) Cryo-EM density map (blue mesh) without B-factor sharpening and the superimposed model of TM. Left, $MTC^{ATP\gamma S}$; right, MTC^{apo}. View orientation and colors as in Figure 4C.

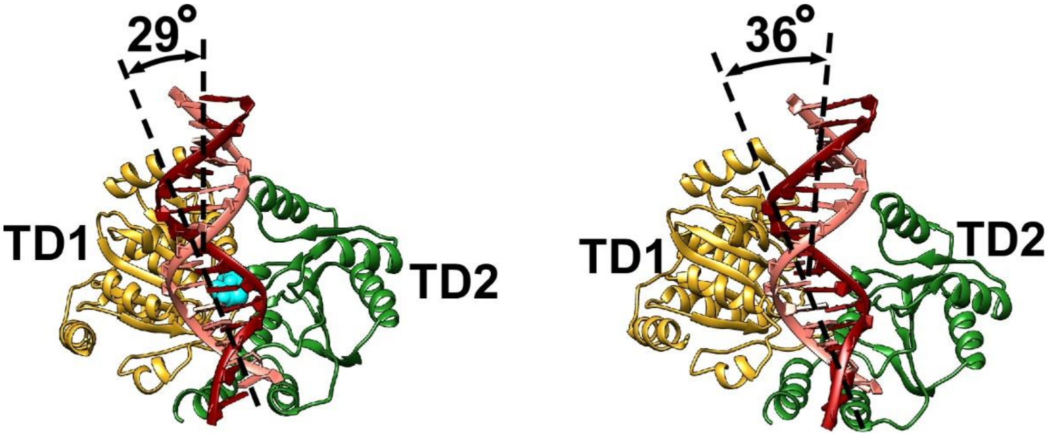

Figure S10. There is a kink in the middle of the upstream dsDNA.
Left, cryo-EM structure of $MTC^{ATP\gamma S}$; right, cryo-EM structure of MTC^{apo}. The DNA distortion angles were measured with UCSF Chimera. The centroid positions were created at sites -25, -16, and -11, and the angle between the centroid positions were measured using the Angle/Torsion function. Protein and nucleic-acid scaffold are shown as ribbon; ATPγS is shown as spheres. Salmon, nontemplate strand DNA; red, template strand DNA; yellow, TD1; green, TD2; cyan, ATPγS.

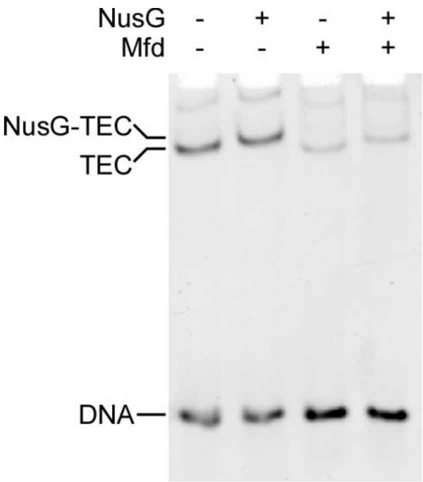

Figure S11. NusG does not interfere with Mfd in RNAP displacement assay.

The concentration of NusG is 5 μM, which is sufficient to bind TEC.

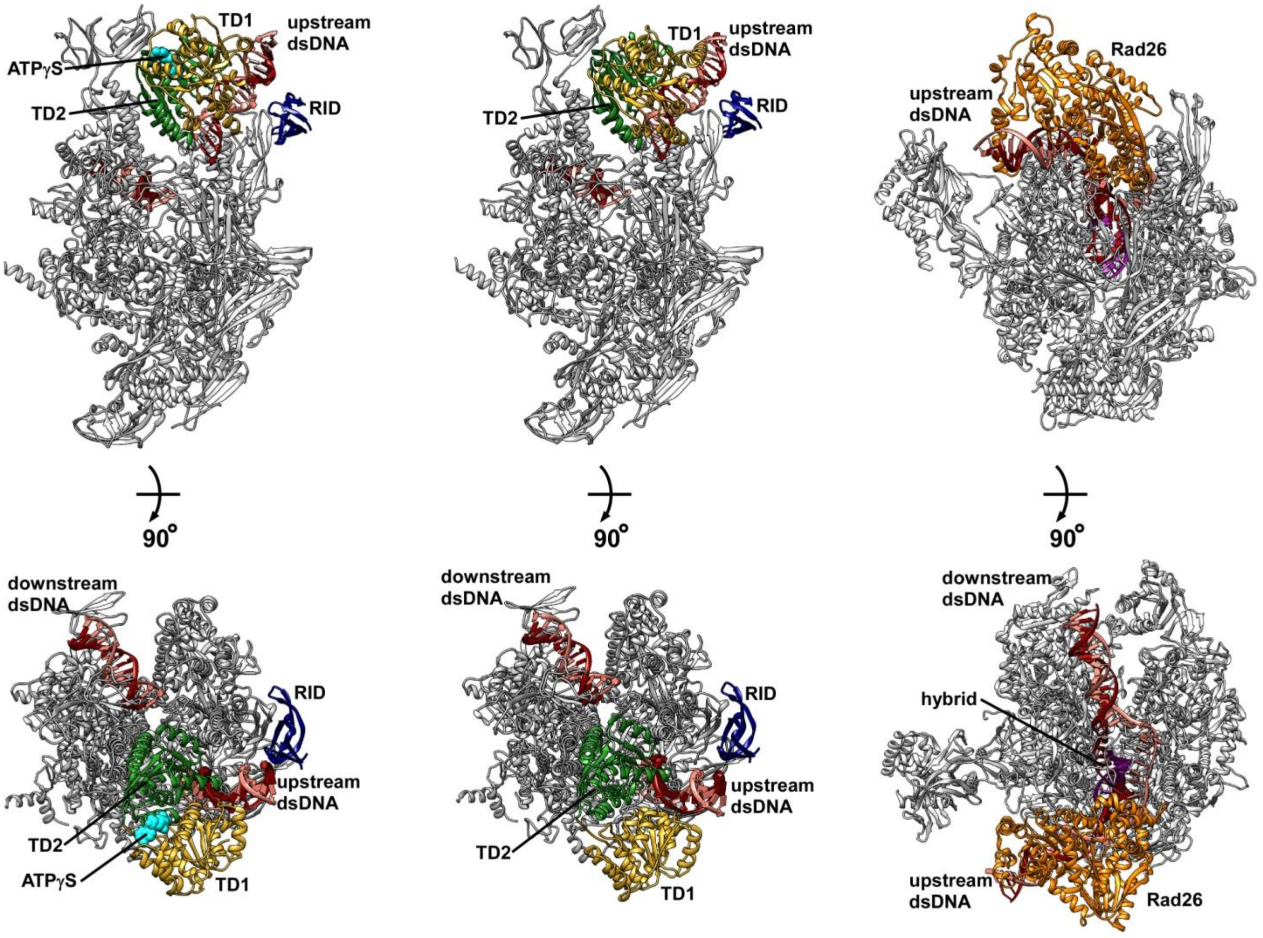

Figure S12. Structural comparison of MTC$^{ATP\gamma S}$ (left), MTC^{apo} (middle), and Rad26-Pol II complex (right, PDB 5VVR).

Gray, RNAP; red, template strand DNA; salmon, nontemplate strand DNA; magenta, RNA; blue, RID; yellow, TD1; green, TD2; cyan, ATPγS; orange, Rad26. View orientations as in Figure 1C.

Table S1. Cryo-EM data collection and refinement statistics.

	MTC$^{ATP\gamma S}$	MTC^{apo}	TEC
**Data collection and processing**			
Microscope	Titan Krios	Titan Krios	Titan Krios
Voltage (kv)	300	300	300
Detector	K2 summit	K2 summit	K2 summit
Electron exposure (e/Å^2)	59	59	59
Defocus range (μm)	1.5-2.5	1.5-2.5	1.5-2.5
Data collection mode	Counting	Counting	Counting
Physical pixel size (Å/pixel)	1.307	1.307	1.307
Symmetry imposed	C1	C1	C1
Initial particle images	661,783	1,149,168	1,149,168
Final particle images	60,650	24,037	558,003
Map resolution (Å)[a]	4.1	5.0	3.1
**Refinement**			
Map sharpening B-factor (Å^2)	-98	-126	-88
Root-mean-square deviation			
Bond lengths (Å)	0.007	0.007	0.003
Bond angles (°)	0.931	0.858	0.539
Molprobity statistics			
Clashscore	10.45	16.27	6.30
Rotamer outliers (%)	0.38	0.79	0.00
Cβ outliers (%)	0.00	0.00	0.00
Ramachandran plot			
Favored (%)	94.49	93.16	98.03
Outliers (%)	0.00	0.00	0.00

[a]Gold-standard FSC 0.143 cutoff criteria.

Supplementary References

1. Robert, X. and Gouet, P. (2014) Deciphering key features in protein structures with the new ENDscript server. *Nucleic Acids Res.*, **42**, W320-W3244.
2. Cardone, G., Heymann, J.B. and Steven, A.C. (2013) One number does not fit all: mapping local variations in resolution in cryo-EM reconstructions. *J. Struct. Biol.*, **184**, 226-236.
